# Evidence that direct inhibition of transcription factor binding is the prevailing mode of gene and repeat repression by DNA methylation

**DOI:** 10.1038/s41588-022-01241-6

**Published:** 2022-12-05

**Authors:** Sebastian Kaluscha, Silvia Domcke, Christiane Wirbelauer, Michael B. Stadler, Sevi Durdu, Lukas Burger, Dirk Schübeler

**Affiliations:** 1 Friedrich Miescher Institute for Biomedical Research, Basel, Switzerland; 2 University of Basel, Faculty of Sciences, Basel, Switzerland; 3 Department of Genome Sciences, University of Washington School of Medicine, Seattle, WA USA; 4 Swiss Institute of Bioinformatics, Basel, Switzerland; 5 Present Address: University of Basel, Faculty of Sciences, Basel, Switzerland

**Keywords:** Gene regulation, Epigenetics

## Abstract

Cytosine methylation efficiently silences CpG-rich regulatory regions of genes and repeats in mammalian genomes. To what extent this entails direct inhibition of transcription factor (TF) binding versus indirect inhibition via recruitment of methyl-CpG-binding domain (MBD) proteins is unclear. Here we show that combinatorial genetic deletions of all four proteins with functional MBDs in mouse embryonic stem cells, derived neurons or a human cell line do not reactivate genes or repeats with methylated promoters. These do, however, become activated by methylation-restricted TFs if DNA methylation is removed. We identify several causal TFs in neurons, including ONECUT1, which is methylation sensitive only at a motif variant. Rampantly upregulated retrotransposons in methylation-free neurons feature a CRE motif, which activates them in the absence of DNA methylation via methylation-sensitive binding of CREB1. Our study reveals methylation-sensitive TFs in vivo and argues that direct inhibition, rather than indirect repression by the tested MBD proteins, is the prevailing mechanism of methylation-mediated repression at regulatory regions and repeats.

## Abstract (web summary)

Co-deletion of all proteins with a functional methyl-CpG-binding domain does not impact genes or repeats silenced by DNA methylation. Analysis of chromatin accessibility and sequence motifs identifies transcription factors that appear sensitive to DNA methylation.

## Main

Over 80% of cytosines in the context of CpG dinucleotides are methylated in mammalian genomes. Methylation of CpG-dense promoters causes stable transcriptional repression^1,2^ and is the basis for long-term monoallelic silencing^3^, such as X chromosome inactivation and genomic imprinting^4^. DNA methylation is also associated with silencing of retrotransposons in somatic tissues^5^ and tumor suppressor genes in cancer^1^.

Two pathways, which are not mutually exclusive, are presumed to be responsible. The first operates in an indirect manner, via proteins that recognize methylated CpGs, as first shown for the MBD of MeCP2 (ref. ^6^). Based on homology, four additional MBD proteins (MBD1–4) as well as six proteins with an MBD-like domain, also known as a TAM (TIP5/ARBP/MBD) domain, were discovered^7–9^; the latter, however, did not bind methylated DNA^10,11^. Only four MBD proteins harbor a functional domain that binds methylated DNA in vitro and in vivo: MeCP2, MBD1, MBD2 and MBD4 (refs. ^6,9,12^). MBD3 harbors a mutated MBD^13^, which does not locate to methylated sequences in the genome^9,12–14^ and is not required for its function^15^. Other factors bind methylated DNA via structurally divergent domains yet require additional sequence context or are limited to hemimethylated DNA^16^. In contrast, 5mC-binding MBD proteins recognize symmetrically methylated CpG dinucleotides in a largely sequence-independent fashion^12,16^. Together with protein interaction studies and in vitro experiments, these findings have established a model in which MBD proteins recruit histone deacetylases to methylated DNA, contributing to transcriptional repression^17–21^.

The second mechanism for repression is direct obstruction of TF binding by cytosine methylation within their motif^22^. Although sensitivity of several TFs to methylation of their binding site has been observed in biochemical assays^23–29^, evidence in the cellular context remains scarce^28–30^.

Defining the contribution of both pathways is critical for our understanding of epigenetic silencing in mammals. Loss of individual MBD proteins results in only mild phenotypes in mice^31–33^ with the exception of MeCP2, whose mutation can cause Rett syndrome^19,34,35^. Combinatorial deletions of *Mbd2/MeCP2* (ref. ^19^) or *Mbd2/MeCP2/Kaiso*^36^ in mice did not reveal a pronounced phenotype (other than Rett syndrome). Functional redundancy between MBDs has accordingly been suggested to account for the absence of severe transcriptional upregulation in the single or combinatorial knockouts generated thus far^7,37^. To date, no combined genetic deletion of all four MBD proteins has been reported.

Complete removal of DNA methylation has been achieved by deletion of the DNA methyltransferases (DNMTs) *Dnmt1*, *Dnmt3a* and *Dnmt3b*. This, however, led to rapid cell death in all tested mammalian cell types with the exception of mouse embryonic stem (ES) cells^38–40^. These are derived from preimplantation blastocysts, whose genomes are globally demethylated^41^. Thus, mechanisms are in place to ensure cellular function despite low DNA methylation levels, which are lost in soma^42^. The observed essential nature of DNMTs in other contexts has been attributed to misregulation of critical genes^43^, activation of repeats^44^ or to DNA damage and the resulting mitotic catastrophe^38^.

Here we aimed to tease apart the contribution of direct versus indirect modes of repression by contrasting cells lacking DNA methylation (both modes affected) and those lacking MBDs (only indirect mode affected). We generated cells lacking all four functional MBD proteins, which unexpectedly had only a minor impact on gene expression in both murine stem cells and derived neurons, as well as a human cell line. The absence of DNA methylation, however, activates genes controlled by methylated CpG islands and causes rampant transcription of retrotransposons specifically in neurons. This entails reorganization of the accessibility landscape by TFs that are methylation sensitive, driving both genic and repeat upregulation. Together, these results suggest direct inhibition of TF binding as the prevailing mode of repression of regulatory regions by DNA methylation.

## Results

### ES cells are viable in the absence of 5mC-binding MBD proteins

**Fig. 1:**
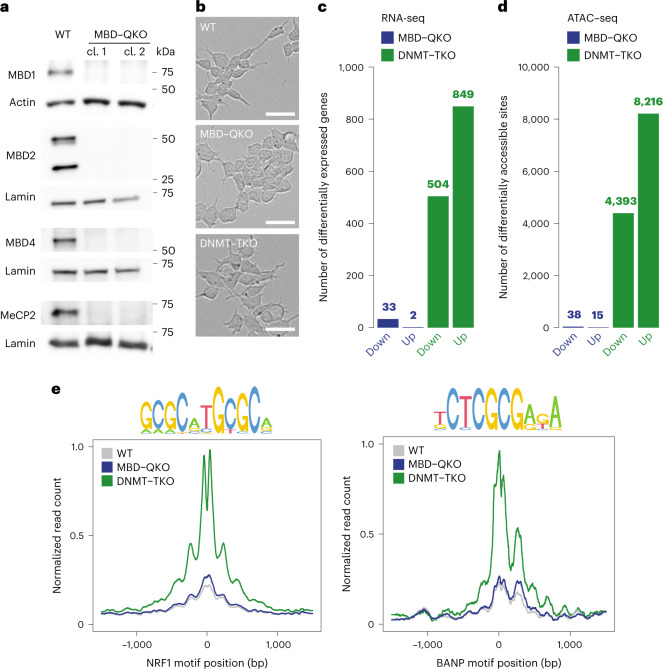
ES cells are viable in the absence of 5mC-binding MBD proteins and display limited changes in transcription and chromatin accessibility. **a**, Detection of MBD proteins by immunoblot in nuclear extracts from WT ES or two MBD–QKO clones (cl. 1 and 2) derived independently with different sets of gRNAs. Actin or lamin serves as loading control. Blots are representative of at least three independent experiments. **b**, Representative images showing morphology of WT, MBD–QKO and DNMT–TKO ES cells. Scale bars, 50 µm. **c**, Number of differentially expressed genes (false discovery rate (FDR) ≤ 0.01 and |log_2_ fold change (FC)| ≥ 1) measured by RNA-seq in mutant ES cell lines compared with WT. **d**, Number of differentially accessible peaks (FDR ≤ 0.01 and |log_2_FC| ≥ 1) as measured by ATAC–seq in mutant ES cell lines compared with WT. **c**,**d**, Replicates from both MBD–QKO clones were combined. **e**, Chromatin accessibility changes in different cell lines at motifs NRF1 (*n* = 745) or BANP (*n* = 13) (indicated by sequence logo on top) that gained accessibility (from **d**) in DNMT–TKO ES cells. ATAC–seq replicates were merged and replicates from both MBD–QKO clones were combined. Source data

**Extended Data Fig. 1:**
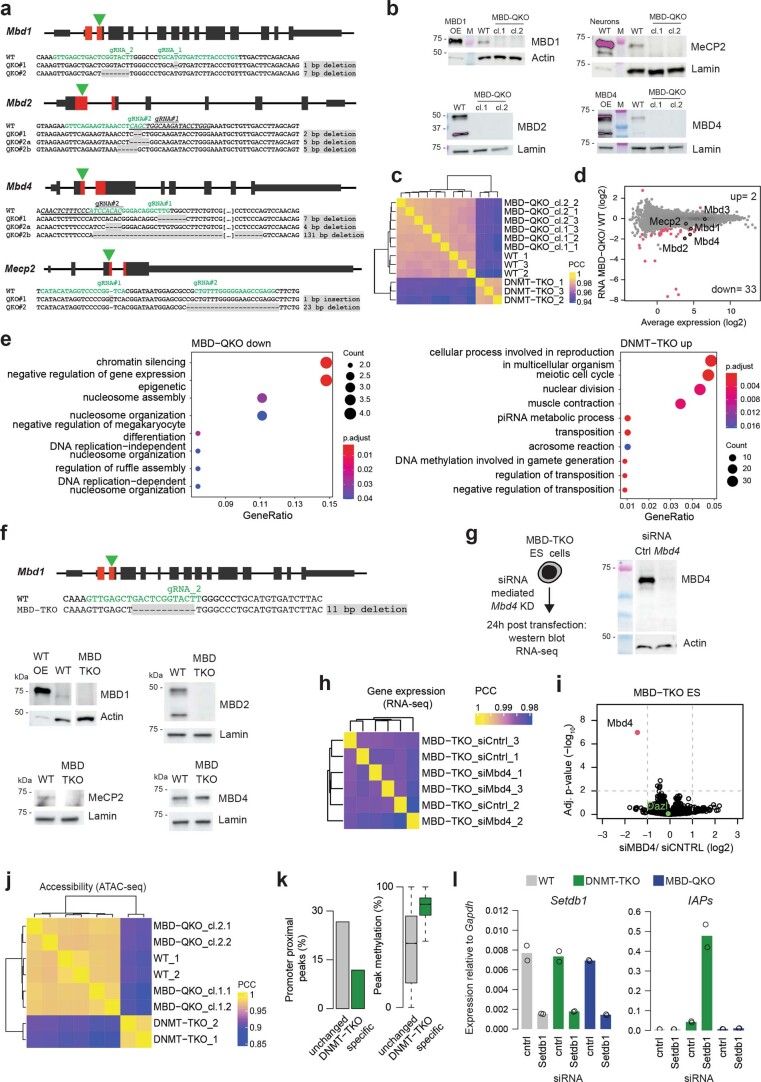
ES cells are viable without 5mC-binding MBD proteins with limited changes in transcription and chromatin accessibility. **a**, MBD sequences in WT and MBD-QKO clones (QKO) #1 and #2. Two sequences shown if the mutation is heterozygous (a and b). Genes with exons (black), gRNA target sites (green triangle) and MBD coding region (red). gRNA sequence highlighted (green or underlined). **b**, Representative Western blot of at least three independent experiments detecting MBD proteins in clones from two independent sets of gRNAs with loading control. Nuclear extracts from ES cells. MBD1 or MBD4 OE: WT ES cells overexpressing (OE) MBD protein from Baubec et al.^12^. WT Neurons: Nuclear extract of WT neurons (Methods). Purple color, signal saturation. M, marker. **c**, Unsupervised clustering of RNA-seq samples from WT and mutant ES cells (RPKM). PCC, Pearson’s correlation coefficient. **d**, Differentially expressed genes (red, FDR = < 0.01 and |log2FC| >= 1) between WT and MBD-QKO ES cells. Replicates from both MBD-QKO clones combined. **e**, Gene Ontology terms enriched among downregulated genes in MBD-QKO (excluding *Mbd* genes, n = 31) or upregulated genes in DNMT-TKO (n = 849) compared to WT (Methods). **f**, Sequence of *Mbd1* for MBD-TKO ES cells (Methods) as in a. Western blots showing absence of MBD1, MBD2 and MeCP2. MBD1 WT OE as in **b**. **g**, Representative Western blot of at least two independent experiments showing MBD4 depletion in MBD-TKO cells after siRNA treatment (24 h). **h**, Unsupervised clustering of RNA-seq samples (RPKM) from MBD-TKO ES cells treated with control or *Mbd4* siRNA. **i**, Number of expression changes (RNA-seq) detected in MBD-TKO cells treated with control or *Mbd4* siRNA (except for *Mbd4)*. **j**, Unsupervised clustering of ATAC-seq samples. Pairwise PCC of log-transformed normalized read counts in peaks indicated. **k**, Features of ATAC-seq peaks that are unchanged between cell lines or that are DNMT-TKO specific. Promoter proximal, distance to TSS < 1000 bp. **l**, RT-qPCR of *Setdb1* and *IAP-gag* relative to *Gapdh* 72 h after transfection of siRNA against *Setdb1* or control. *Setdb1* knockdown causes strong increase in IAP activity only in DNMT-TKO cells. These cells already show moderate IAP activity in control conditions, as previously described^45,74^. Two biological replicates per condition (n = 2) with each measurement representing the mean of three technical replicates. Source data

Because mouse ES cells are viable in the absence of DNA methylation^42,45^, we reasoned that they should be amenable to comprehensive deletions of readers of this epigenetic mark. We focused on MBD1, MBD2, MBD4 and MeCP2 (henceforth MBD proteins) as established 5mC binders in vitro and in vivo^6,9,12^. Using sequential CRISPR targeting we generated two independent mouse ES cell lines, using a different set of guide RNAs, that are a quadruple knockout of these four MBD protein genes (MBD–QKO) as verified by sequencing and immunoblotting (Fig. 1a and Extended Data Fig. 1a,b).

MBD–QKO ES cells are viable in culture, with normal proliferation and morphology (Fig. 1b). Also, at the level of the transcriptome, MBD–QKO ES cells closely resemble wild-type (WT) ES cells (Extended Data Fig. 1c). Only two genes are reproducibly upregulated in both clones while 33 are downregulated (Fig. 1c and Extended Data Fig. 1c–e). This limited transcriptional response is unlikely to be the result of compensatory mechanisms that follow a stronger, transient response because it was also observed following acute depletion of a single remaining MBD by small interfering RNA knockdown in a MBD triple-knockout cell line (MBD–TKO) (Extended Data Fig. 1f–i). To determine genome-wide effects on chromatin accessibility in MBD–QKO cells we performed an assay for transposase-accessible chromatin using sequencing (ATAC–seq). This revealed only minor changes in MBD–QKO, in line with the modest transcriptional response (Fig. 1d and Extended Data Fig. 1j).

To contrast loss of the tested MBD proteins with that of DNA methylation, we deleted *Dnmt1/3a/3b* in ES cells using CRISPR–Cas9 (DNMT–TKO), rendering ES cells free of DNA methylation. In contrast to MBD–QKO ES cells, DNMT–TKO ES cells display gene expression changes at several hundred genes, with 504 down- and 849 upregulated (Fig. 1c). Upregulated genes are enriched for being gamete specific (Extended Data Fig. 1e), because many of these are controlled by CpG-rich promoters that are methylated and silent outside of the germline^46^.

Profiling the chromatin accessibility landscape by ATAC–seq in DNMT–TKO ES cells identified several thousand regions that gain accessibility compared with WT (Fig. 1d). As previously observed by us using DNase-seq^30^, these are methylated in WT, located distally from promoters (Extended Data Fig. 1k) and contain motifs for known methylation-sensitive TFs such as NRF1 (ref. ^30^) or BANP^47^. In cells lacking MBD proteins, these sites do not gain accessibility (Fig. 1e).

In summary, while ES cells tolerate the loss of either MBD proteins or DNMTs, only the absence of DNA methylation substantially perturbs the transcriptome and genomic accessibility.

### Neuronal transcriptomes in the absence of DNMT or MBD proteins

DNA methylation-independent pathways, such as trimethylation of lysine 9 of histone H3 (H3K9me3), which is mediated by SETDB1 and targeted via KRAB-ZNF proteins, are thought to account for repeat repression in DNMT–TKO ES cells^42,45,48–53^. As a direct test of whether this could similarly mask potential functions of MBD proteins, we reduced SETDB1 levels by siRNA transfection in WT, DNMT–TKO and MBD–QKO ES cells (Extended Data Fig. 1l). We then monitored expression levels of evolutionary young intracisternal A-type particle (IAP) repeats^54^ (Extended Data Fig. 1l), which showed strong upregulation only in DNMT–TKO cells and not in MBD–QKO or WT cells^45^. Thus, the absence of DNA methylation but not of MBD proteins causes increased sensitivity to levels of SETDB1.

**Fig. 2:**
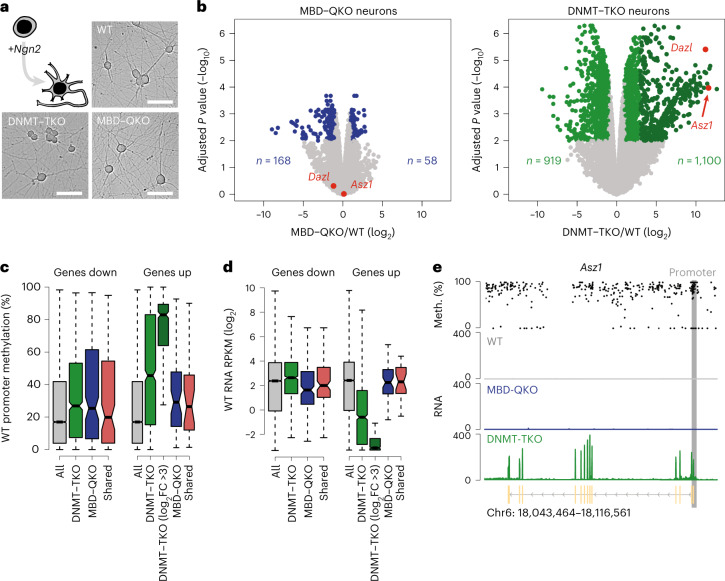
Neurons lacking MBD or DNMT proteins display distinct transcriptional phenotypes. **a**, ES cells carrying an inducible *Ngn2* expression cassette can be rapidly differentiated towards neurons. Images represent morphology of WT, MBD–QKO and DNMT–TKO neurons 8 days after induction for at least three independent differentiation experiments. Scale bars, 50 µm. **b**, Gene expression changes between mutant and WT neurons; left, MBD–QKO; right, DNMT–TKO. Differentially expressed genes are depicted in blue or green (FDR ≤ 0.01 and |log_2_FC| ≥ 1); genes that strongly gained expression in DNMT–TKO (FDR ≤ 0.01 and log_2_FC > 3) are depicted in dark green (*n* = 434) and germline-specific genes *Dazl* and *Asz1* in red. **c**,**d**, Average promoter methylation levels (**c**) and gene expression levels (**d**) in WT neurons of all expressed genes (total, *n* = 15,452) or of all genes differentially expressed in MBD–QKO and DNMT–TKO neurons (from **b**) or those shared between both conditions (shared: down *n* = 111, up *n* = 27). Black lines, median; boxes, first and third quartile; whiskers, maximum and minimum values of distribution after removal of outliers (Methods). **e**, Single-locus example of a germline-specific gene (*Asz1*) with a methylated promoter that is derepressed in the absence of DNA methylation (meth.) but not in the absence of MBD proteins. RPKM, reads per kilobase million.

**Extended Data Fig. 2:**
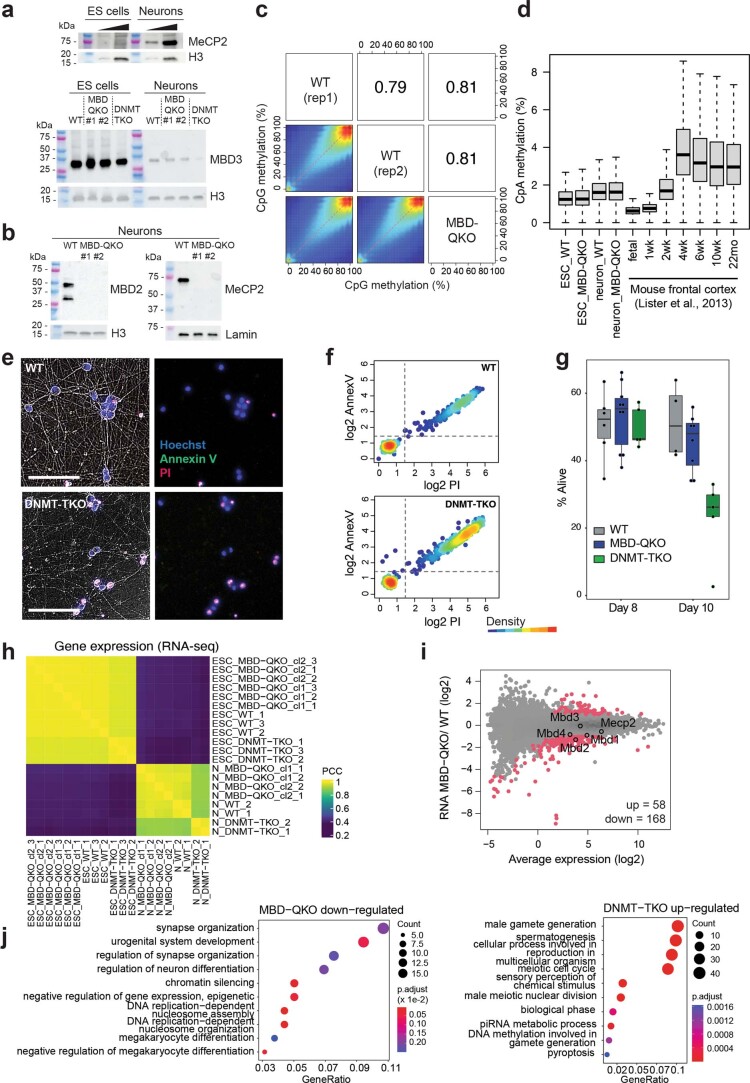
Neurons without DNMT or MBD proteins show distinct phenotypes. **a**, NGN2 driven neuronal differentiation coincides with higher MeCP2 levels^92^ and lower MBD3 levels^12^ as shown by Western blot detection in nuclear extracts. Blots representative examples of at least two independent experiments. **b**, Western blot for MBD2 and MeCP2 validating their absence in MBD-QKO neurons. No signal was detected for MBD1 and MBD4 in WT and MBD-QKO neurons. Blots representative for at least two independent experiments. **c**, CpG methylation in neurons between two WT replicates and one MBD-QKO sample (down-sampled to the sample with lowest coverage and retaining only CpGs with ten-fold coverage). **d**, CpA methylation in WT and MBD-QKO ES and NGN2 derived neurons compared to the mouse frontal cortex of different developmental stages^66^. Boxplots as in Fig. 2d. **e**, Representative brightfield (inverted) and fluorescent composite images of WT and ten days DNMT-TKO neurons of at least four regions from two independent experiments. Live staining with Hoechst (blue) and cell death marked by AnnexinV (green) and propidium iodide (PI, red). Scale bar = 100 µm. **f**, Quantification of survival in neurons, measured by AnnexinV and PI signal intensities on nuclear Hoechst segmented nuclei. Cutoffs indicated by gray dashed lines. **g**, Boxplot of cell viability. n = cells examined over N randomly chosen regions. Day eight: WT n = 849, N = 6; MBD-QKO n = 1433, N = 10; DNMT-TKO n = 992, N = 5, day ten: WT n = 509, N = 4; MBD-QKO n = 1066, N = 8; DNMT-TKO n = 1086, N = 5. Replicates were combined. Boxplots as in Fig. 2d. **h**, Unsupervised clustering of RNA-seq samples from WT and mutant ES and neuron cells (RPKM). Colors indicate PCC. **i**, Differentially expressed genes (red, FDR = < 0.01 and |log2FC | >= 1) in MBD-QKO neurons vs WT. *Mbd* genes, black circles. Replicates from both MBD-QKO clones were combined. **j**, Gene Ontology (GO) terms enriched in the set of genes downregulated in MBD-QKO neurons (n = 168) or upregulated in DNMT-TKO neurons (n = 1100) compared to WT. The dots represent the top terms with highest gene ratio (fraction of genes represented in the given GO term) with dot size and color representing gene counts and the adjusted p-value (Fisher’s exact test), respectively. Source data

Next we reasoned that a repressive role of MBD proteins might be evident only in differentiated cells, where DNA methylation becomes essential^38,40,55–59^. Testing this is hindered by the fact that DNMT–TKO cells do not differentiate when using a classic, several-week-long protocol to generate neurons^42,60^ (data not shown), in line with the observation that DNA hypomethylation in the adult brain causes lethality in neurons^61,62^. We speculated that a rapid differentiation regime might enable generation—at least for a limited time—of methylation-free neuronal cells: ectopic expression of a neurotrophic TF (NGN2) produces functional glutamatergic neurons within a few days^63,64^. The parental ES cell line from which all clones were derived harbors a dox-inducible *Ngn2* expression cassette. Following induction of *Ngn2*, both DNMT–TKO and MBD–QKO cells exited the cell cycle, adopted neuronal morphology and formed axonal networks similar to WT within about 3 days (Fig. 2a and Extended Data Fig. 2a,b).

Absence of the tested MBD proteins has no detectable effect on genomic patterns of CpG methylation (Extended Data Fig. 2c). Neurons derived from both genotypes show increased CA methylation^65–67^ but at levels lower than previously observed in adult mouse brain (Extended Data Fig. 2d), probably reflecting the limited culturing time^66^.

Thus, neuronal cells can be generated in vitro in the absence of DNA methylation or MBD proteins using a rapid neurogenesis paradigm, allowing us to study the effects on genome regulation in a differentiated and postmitotic cell state. While the absence of MBD proteins did not affect the long-term viability of the derived neurons, DNMT–TKO neurons showed decreased viability at around 10 days following induction (Extended Data Fig. 2e–g).

The transcriptome of MBD–QKO neurons is remarkably similar to that of WT neurons, with minor but reproducible changes (168 genes down, 58 genes up) (Fig. 2b and Extended Data Fig. 2h,i). Affected genes tend to have unmethylated promoters (Fig. 2c), are already active in WT neurons (Fig. 2d) and are enriched in different pathways of tissue development (Extended Data Fig. 2j), implying that loss of MBD-mediated indirect repression at methylated regions is not the primary driver of these changes.

The transcriptome of DNMT–TKO neurons resembles that of WT neurons (Extended Data Fig. 2h), indicating that they acquire a neuronal identity in line with their morphology. However, they are more dissimilar to WT than MBD–QKO neurons, displaying a roughly tenfold larger set of differentially expressed genes (Fig. 2b). Genes upregulated in DNMT–TKO neurons tend to be under the control of promoters that are methylated (Fig. 2c), inactive in WT (Fig. 2d) and are again enriched for gamete-specific genes (Extended Data Fig. 2j). Prominent examples include *Dazl* and *Asz1*, which are known to rely on promoter methylation for repression in somatic cells^46,68^. These genes are not upregulated in the absence of MBD proteins (Fig. 2b,e), arguing against a prominent role of the tested MBD proteins in maintenance or establishment of repression of genes with methylated promoters that become activated in the absence of DNA methylation in neurons.

### Limited derepression is conserved in human MBD–QKO cells

**Extended Data Fig. 3:**
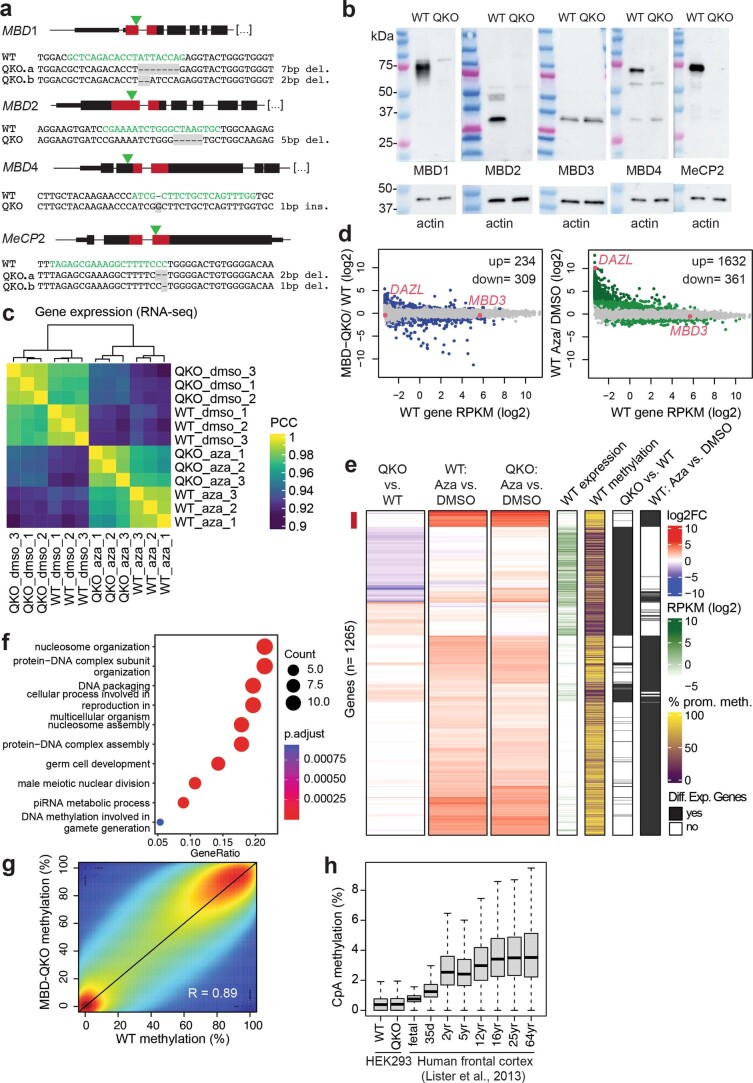
Human HEK293 cells lacking MBD proteins show limited changes in transcription and global levels of DNA methylation. **a**, Sequence of MBD loci in in HEK293 annotated as in Extended Data Fig. 1. **b**, Western blot for MBDs indicating absence of proteins. MBD3 protein levels unchanged (actin represents loading control). Blots representative of at least three independent experiments. **c**, Unsupervised clustering of RNA-seq (RPKM) samples from WT and MBD-QKO HEK293 cells treated with DMSO or 5-Aza-2′- deoxycytidine (Aza). **d**, Genes differentially expressed (FDR = < 0.01 and |log2FC| >= 1) are colored in blue or light green. Strongly upregulated genes in WT cells upon Aza treatment are colored in dark green (FDR = < 0.01 and log2FC > = 3). Comparing WT and MBD-QKO cells reveals that ~ 7-times more genes are upregulated upon Aza treatment, which tend to be transcriptionally silent in DMSO conditions. **e**, Hierarchical clustering of genes differentially expressed in MBD-QKO cells or in WT cells treated with Aza (blue or dark green points in d, n = 1265). Each row depicts the expression fold change (log2) of either: MBD-QKO vs. WT, WT treated with Aza vs. DMSO, MBD-QKO treated with Aza vs. DMSO. WT gene expression, WT promoter methylation and differentially expressed genes (DEG in black, FDR = < 0.01 and |log2FC| >= 1) are indicated. Of all genes that respond to Aza treatment in WT cells, only ~6 % (n = 20) of down- and 7 % (n = 110) of upregulated genes are also affected in MBD-QKO cells. The gene cluster with most upregulated genes under Aza treatment is marked with a red bar. These genes are largely unaffected after deletion of MBD proteins and are only de-repressed in MBD-QKO cells when treated with Aza. **f**, Gene ontology enrichment of genes represented in the cluster indicated by the red bar in e (Methods). **g**, Methylation frequencies in 1 kb windows comparing WT and MBD-QKO HEK293 cells (min. read coverage of 10 in both samples, n = ~2 mio). **h**, CpA methylation levels of 1 kb windows in HEK293 cells or human frontal cortex from Lister et. al.^66^ (windows with min. read coverage of 100 in all samples). Boxplots as in Fig. 2d.

Before further exploring the molecular consequences of the absence of MBD proteins in neurons, we asked whether the MBD–QKO phenotype is conserved in human cells. To do so we generated a MBD–QKO from human HEK293 cells (Extended Data Fig. 3a,b). These are viable and, again, show only a limited number of genes to be misregulated (down, 309; up, 234; Extended Data Fig. 3c,d), similar to the murine system. To generate hypomethylated cells we treated WT HEK293 cells with the DNMT1 inhibitor 5-Aza-2′-deoxycytidine (Aza), which reduced global methylation from 70 to 20% (ref. ^69^). Again more genes change expression than in the MBD–QKO (Extended Data Fig. 3d); upregulated genes tend to have a methylated promoter, are transcriptionally inactive in the absence of the compound and are again enriched for germline genes, including *DAZL* (Extended Data Fig. 3d–f). In contrast, genes differentially expressed in MBD–QKO cells are already transcriptionally active and show low promoter methylation in WT (Extended Data Fig. 3e). Genome-wide methylation levels of MBD–QKO cells are comparable to those of WT HEK293 cells (Extended Data Fig. 3g) which, unlike the murine system, have virtually no CpA methylation (Extended Data Fig. 3h). This suggests that, in this human cell line, DNA methylation-mediated repression can occur only in the context of CpG yet is independent of 5mC-binding MBD proteins.

### Accessibility changes following loss of DNMT, but not MBD, proteins

**Fig. 3:**
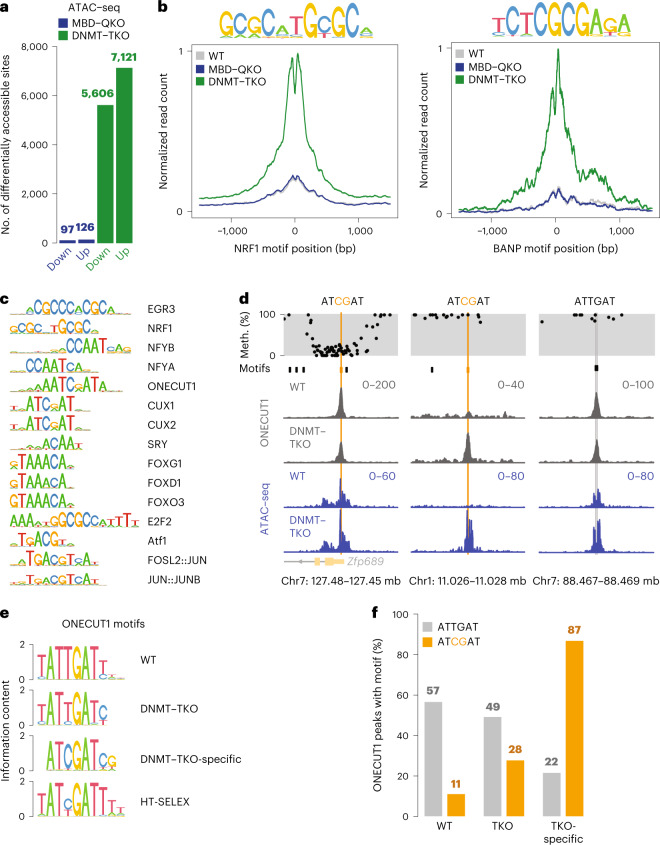
Loss of DNA methylation, but not the tested MBD proteins, reshapes the chromatin accessibility landscape revealing methylation-sensitive TFs in neurons. **a**, Differentially accessible peak regions (FDR < 0.01 and |log_2_FC| > 1) identified by ATAC–seq in mutant neurons compared with WT. Replicates from both MBD–QKO clones were combined. **b**, Accessibility changes at motifs of NRF1 (*n* = 792) and BANP (*n* = 26) that gained accessibility in DNMT–TKO neurons. For each condition at least two replicates were combined. **c**, Unbiased clustering of the top 15 motifs enriched in DNMT–TKO-specific ATAC–seq peaks by weight matrix similarity (FDR < 0.01 and |log_2_FC| > 1). **d**, Single-locus examples of ONECUT1 binding (gray tracks) in WT and DNMT–TKO neurons at canonical motifs (black) or CpG-containing motif variants (orange). Top track indicates CpG methylation, ATAC–seq tracks in blue. Read counts in running windows of 51 nt (ChIP–seq) or 11 nt (ATAC–seq), replicate data combined. **e**, Motifs identified in the top 500 WT-, DNMT–TKO- or DNMT–TKO-specific (from Extended Data Fig. 6b) ONECUT1 ChIP–seq peak regions compared with a human ONECUT1 motif identified in vitro by HT-SELEX^107^. **f**, ONECUT1 peak regions with canonical or CpG-containing motifs in WT-, DNMT–TKO (TKO)- or TKO-specific peak regions.

**Extended Data Fig. 4:**
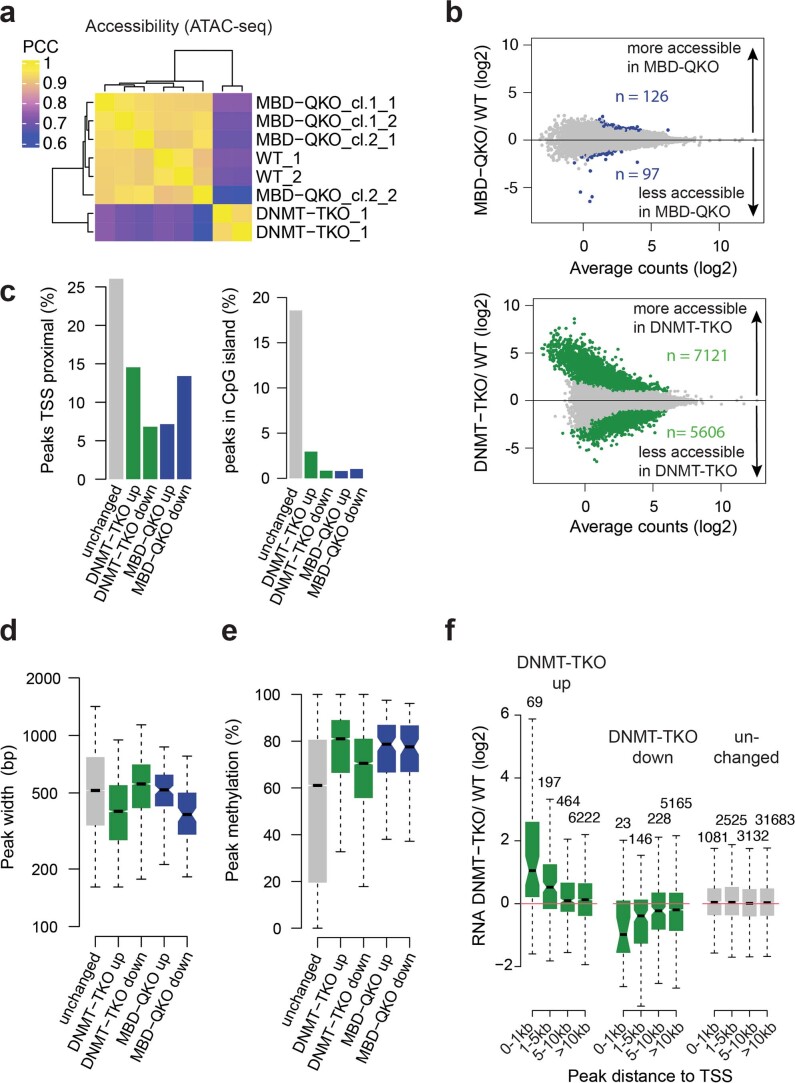
The chromatin accessibility landscape in neurons changes in response to loss of DNA methylation but not MBD proteins. **a**, Unsupervised clustering of ATAC-seq samples from WT and mutant neuron cells. Colors indicate pairwise Pearson’s correlation coefficients (PCC) of log-transformed normalized read counts in ATAC-seq peaks, indicating clear separation of DNMT-TKO from WT and MBD-QKO ES cells. **b**, MA plot showing mean chromatin accessibility (ATAC-seq) versus accessibility changes for neurons lacking MBD proteins or DNA methylation compared to WT. For differential accessibility analysis all replicates from both MBD-QKO clones were combined. Sites with an |log2FC| > 1 compared to WT and FDR < 0.01 are colored. **c**, Percent TSS-proximal ATAC-seq peaks (<1000 bp, left plot) or peaks overlapping with CpG islands (right plot) from b that do not change in any condition (unchanged, n = ~83,000), or change accessibility in DNMT-TKO (up, n = 7121 or down, n = 5606) or MBD-QKO neurons (up, n = 126 or down, n = 97). **d**, same as in c for ATAC-seq peak width or **e**, ATAC-seq peak methylation levels. Boxplots as in Fig. 2d. **f**, Expression change of genes closest to peaks that are unchanged between conditions or peaks that gain (up) or lose accessibility (down) in DNMT-TKO neurons binned by distance to TSS. Number of peaks per bin is indicated. Boxplots as in Fig. 2d.

Having observed the similar phenotype in human cells, we proceeded to study changes in chromatin in differentiated mouse cells. The neurons showed few accessibility changes in the absence of MBD proteins (Fig. 3a and Extended Data Fig. 4a,b), while DNMT–TKO neurons showed several thousand differentially accessible regions (Fig. 3a and Extended Data Fig. 4b). The majority of sites gain accessibility, tend to locate distally from transcription start sites, are shorter than shared sites and are methylated (Extended Data Fig. 4c–e). Increased accessibility correlates with local transcriptional upregulation (Extended Data Fig. 4f). As in ES cells, known methylation-sensitive NRF1 and BANP sites gain accessibility only in the absence of DNA methylation, but not MBD, proteins (Fig. 3b). We conclude that the absence of DNA methylation, but not MBD proteins, leads to increased accessibility of regulatory regions and upregulation of neighboring genes in neurons, suggesting a contribution of methylation-sensitive TFs.

### Identification of candidate methylation-sensitive TFs

**Extended Data Fig. 5:**
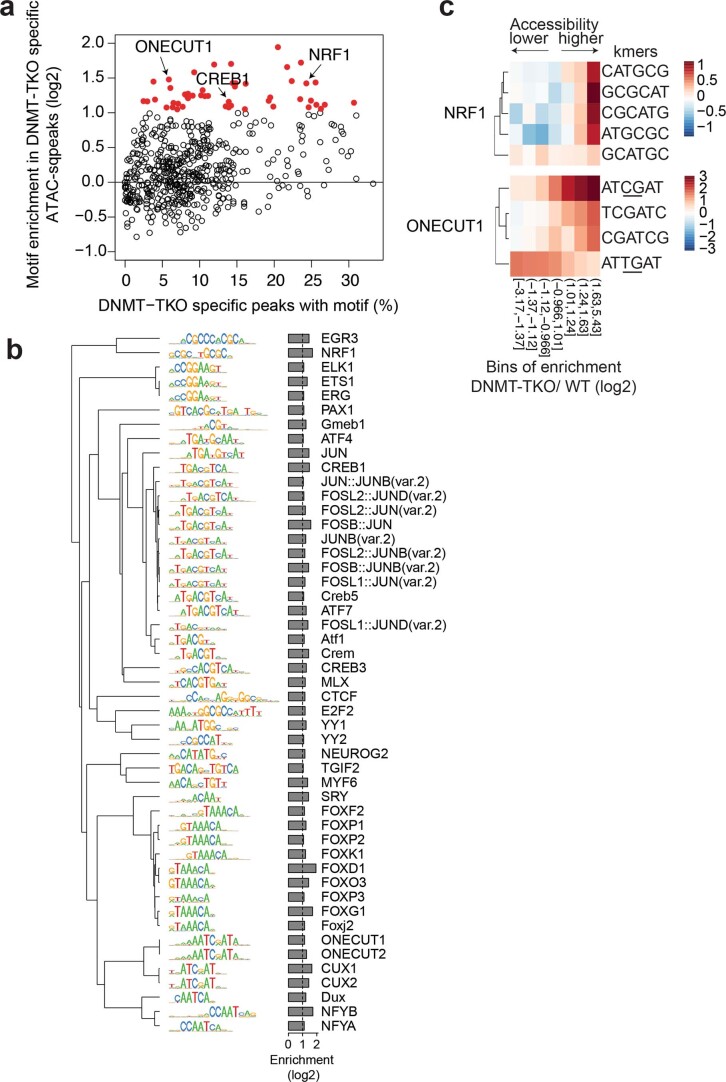
Motif search in DNMT-TKO specific ATAC-seq peak regions. **a**, Motif enrichment (Methods) among DNMT-TKO specific ATAC-seq peaks (FDR < 0.01 and |log2FC| > 3) vs. DNMT-TKO specific peaks with that motif in percent. Red points indicate motifs that are enriched (FDR < 0.01 and |log2FC| > 1) in DNMT-TKO specific peaks (n = 49). **b**, Unbiased clustering of motif similarities (position weight matrices) of motifs enriched in DNMT-TKO specific peaks colored in red in a. **c**, Enrichment (log2) of NRF1 or ONECUT1 hexamers in bins of differentially accessible ATAC-seq peaks between DNMT-TKO and WT neurons (Methods). Similar to NRF1, CpG-containing ONECUT1 hexamers are enriched in bins of peaks that gain accessibility in DNA methylation-deficient cells, opposed to the canonical (CpG-free) ONECUT1 hexamers.

The top DNMT–TKO-specific ATAC–seq peaks in neurons are enriched for 49 known TF motifs (Extended Data Fig. 5a), several with high sequence similarity (Extended Data Fig. 5b). Among those motifs strongly enriched is the one for the methylation-sensitive TF NRF1 (Fig. 3c and Extended Data Fig. 5a) that is ubiquitously expressed^30^. Other prominent motifs are specific to neurons, such as ONECUT1 (also known as HNF6) (Fig. 3c and Extended Data Fig. 5a). Of note, several enriched motifs do not contain a CpG, indicating that they potentially respond to non-CpG methylation despite its low prevalence in our experimental system (Extended Data Fig. 2d). More probably, however, these might not be directly linked to DNA methylation, highlighting the general need for further experimental validation.

### ONECUT1 is a methylation-sensitive TF

**Extended Data Fig. 6:**
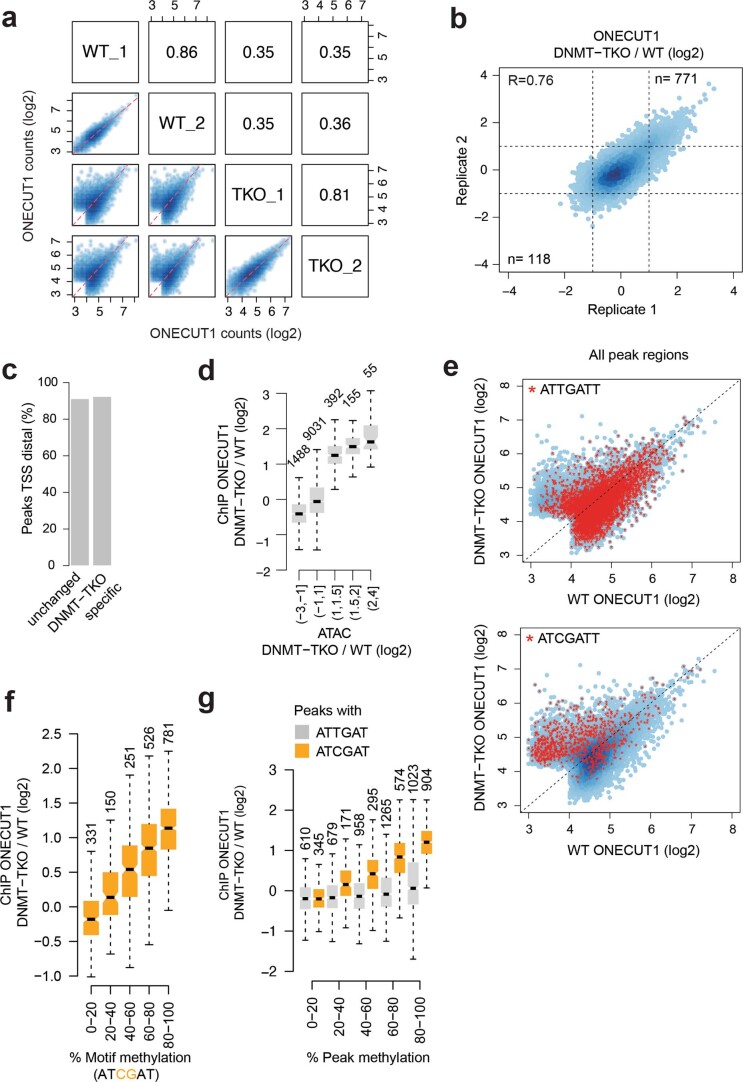
ONECUT1 is methylation-sensitive at its CpG-containing motif variant. **a**, Reproducibility of counts for two independent ONECUT1 ChIP-seq replicates from WT and DNMT-TKO neurons in merged WT and DNMT-TKO peak regions. Pearson correlation coefficients are indicated. **b**, Reproducibility of changes in ONECUT1 binding in DNMT-TKO versus WT neurons in all peaks regions. Number of regions that de- or increase in binding reproducibly are indicated by n. Pearson correlation coefficient is shown **c**, Percent of ONECUT1 peak regions distal (>1000 bp) to the transcriptional start site (TSS). Unchanged, peaks unchanged (log2FC < 1, n = 10,232) between WT and DNMT-TKO neurons or DNMT-TKO specific (n = 771, as defined in **a**.). **d**, Changes of ONECUT1 binding versus changes in chromatin accessibility (ATAC-seq) between WT and DNMT-TKO neurons in all peak regions (n = 11,121). Boxplots as in Fig. 2d. **e**, WT and DNMT-TKO ONECUT1 ChIP-seq signal in all peak regions. Red asterisks mark peak regions that contain the canonical ONECUT1 motif (top) or the CpG-containing variant (bottom) at least once. **f**, Change in ONECUT1 binding between DNMT-TKO and WT at all peak regions grouped according to their WT motif methylation. Boxplots as in Fig. 2d. **g**, Change in ONECUT1 binding between DNMT-TKO and WT peak regions grouped according to peak methylation, split by motif category. Grey box plots show peak regions containing the canonical motif at least once. Orange box plots show peak regions containing the CpG-containing motif variant at least once. Number above box plots indicates the number of peaks. Boxplots as in Fig. 2d.

We first tested ONECUT1, a key regulator of the nervous system, liver and pancreas^70^. Its canonical motif has no CpG yet a variant motif does^71,72^, which is enriched in DNMT–TKO-specific open regions (Extended Data Fig. 5c). Indeed, ONECUT1 binds to ~700 additional sites in DNMT–TKO neurons while only ~100 display slightly reduced binding (Fig. 3d and Extended Data Fig. 6a,b). Newly bound sites reside distally to transcription start sites (Extended Data Fig. 6c), gain accessibility (Fig. 3d and Extended Data Fig. 6d) and are enriched for the variant motif (Fig. 3e,f and Extended Data Fig. 6e). DNMT–TKO-specific peaks that contain the CpG-variant are methylated in WT neurons and show the largest increase in binding at motifs with the highest methylation (Extended Data Fig. 6f). Methylation levels of CpGs in the vicinity of the canonical motif do not correlate with differential binding in DNMT–TKO (Extended Data Fig. 6g), leading us to conclude that ONECUT1 is methylation sensitive in vivo but only at the CpG-containing motif variant. Thus new tissue-specific TFs can be identified by generation of postmitotic cells lacking DNA methylation.

### DNA methylation-dependent derepression of repeats in neurons

**Fig. 4:**
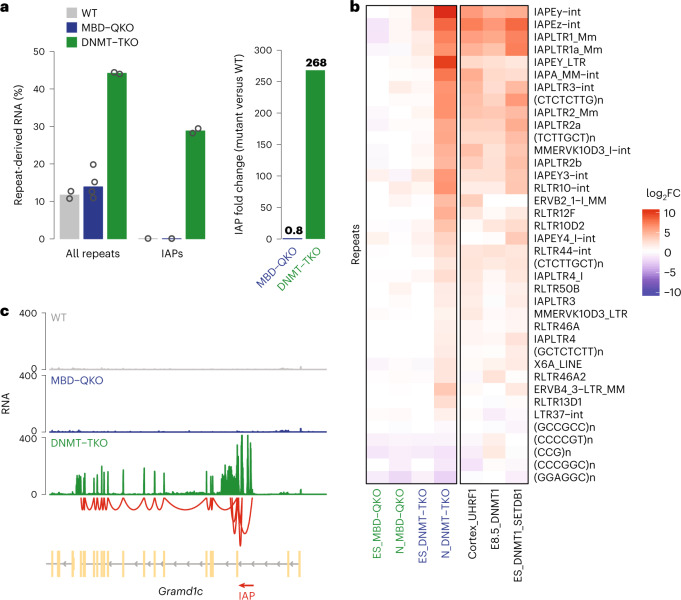
Repeats are derepressed in neurons in the absence of DNA methylation but not in the absence of the tested MBD proteins. **a**, Percentage of reads (all repeats plus genes) mapping to all repeats or IAPs. Numbers of replicates (circles), WT (*n* = 2), MBD–QKO (*n* = 4, both clones combined), DNMT–TKO (*n* = 2). Multimapping reads counted in repeats (Methods). Bar plot shows the median of replicates. **b**, Expression change of repeat subfamilies in mutants over WT cell lines or tissue. Rows depict repeat subfamilies differentially expressed in MBD–QKO or DNMT–TKO neurons (RNA-seq, FDR ≤ 0.01 and |log_2_FC| ≥ 3, with a minimum of ten repeat instances; Extended Data Fig. 7a,b). Left, *Ngn2* mouse ES cells (ES) or derived neurons (N). Right, public RNA expression data: ES_DNMT1_SETDB1, conditional deletion of SETDB1 and DNMT1 in ES cells^74^; Cortex_UHRF1, conditional deletion of Uhrf1 in cerebral cortex of postnatal day 5 mice^61^; E8.5_DNMT1, *Dnmt1* mutant embryos at day 8.5 (ref. ^68^). Multimapping RNA-seq reads were considered (Methods). **c**, Representative gene (*Gramd1c*) activated in DNMT–TKO neurons by a transcribed IAP element (red arrowhead). Red line indicates splice junctions, illustrating that RNA reads derived from the IAP repeat overlap with the downstream exon. Chr16: 43970350−44073345.

**Extended Data Fig. 7:**
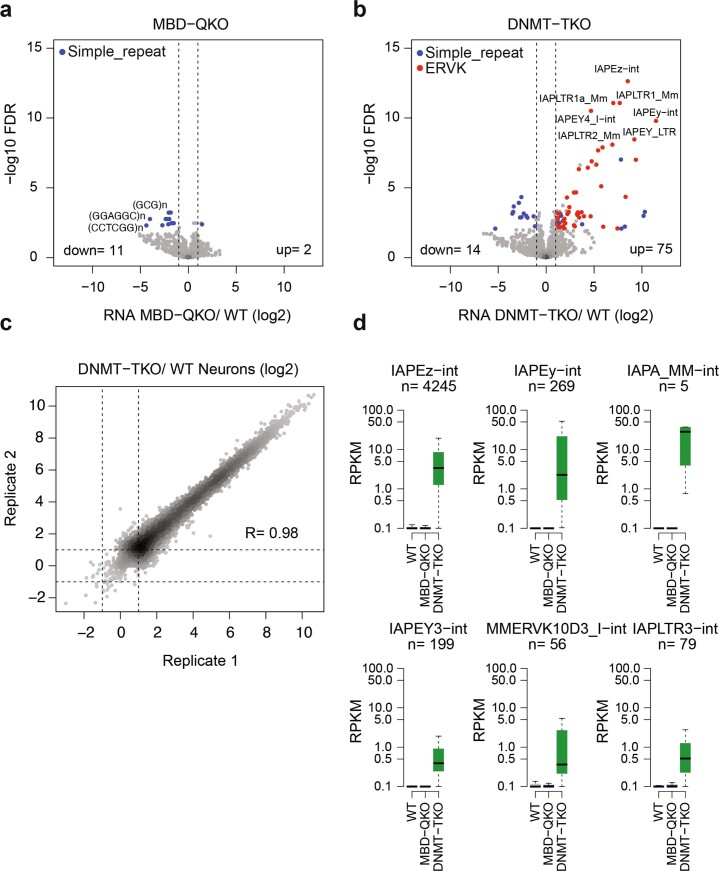
Repeats are de-repressed in absence of DNA methylation but not MBD proteins in neurons. **a**, Volcano plot showing differentially expressed repeat subfamilies in MBD-QKO neurons using random assignment of multi mapping reads. Dashed lines indicate two-fold expression change. Repeat subfamilies belonging to ERVK or simple repeats that are differentially expressed (FDR = < 0.01 and |log2FC| >= 1) are colored. **b**, Same as in a but for DNMT-TKO neurons. **c**, Reproducibility of IAP expression changes in DNMT-TKO versus WT neurons in all IAP elements annotated by RepeatMasker. Only uniquely mapping RNA-seq reads and elements with more than 8 counts in at least one condition are considered. **d**, RNA expression of the top six most significant differentially expressed internal sequence of class-2 endogenous retroviruses from b. Only uniquely mapping RNA-seq reads were used. Internal IAP elements selected to have more than 8 counts in at least one condition. Boxplots as in Fig. 2d.

When asking whether repeat repression is affected in neurons, we observed no significant increase in the absence of MBD proteins (Fig. 4a and Extended Data Fig. 7a). Removal of DNA methylation dramatically increased repeat-derived RNA, in particular from IAP elements, in DNMT–TKO neurons (Fig. 4a,b and Extended Data Fig. 7b). Due to this 200-fold induction (Fig. 4a), IAPs comprise one-third of repeat-derived RNA, which impacts the expression of neighboring genes^68,73^ (Fig. 4c) and is also evident at individual IAP retrotransposons (Extended Data Fig. 7c,d). A comparable derepression has previously been observed in *Dnmt1*^−/−^ ES cells conditionally depleted of SETDB1 (ref. ^74^), in murine *Dnmt1*^−/−^ embryos^5,68^ and in conditional UHRF1-depleted postnatal mouse cortex^61^ (Fig. 4b), suggesting that differentiated neurons in culture recapitulate the upregulation observed in vivo.

### CRE is critical for IAPLTR1/1a activity

**Fig. 5:**
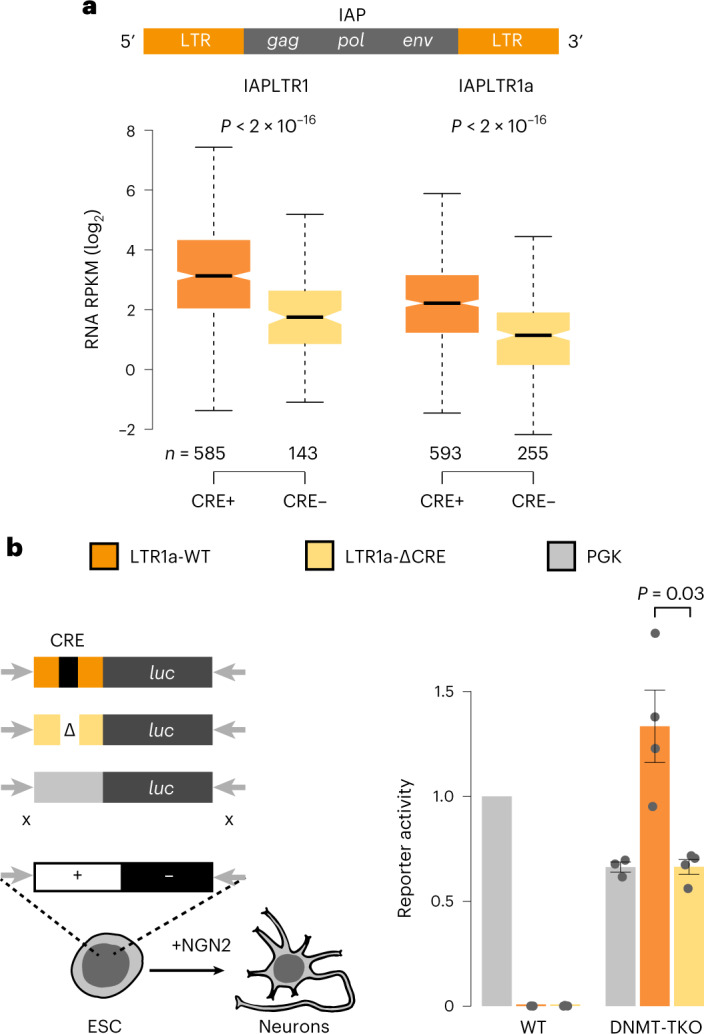
CRE is a feature of upregulated IAPLTR1/1a and important for repeat activity. **a**, Scheme of IAP element and expression of IAPLTR1 or IAPLTR1a elements in DNMT–TKO neurons grouped by the presence of a perfect match with the CRE motif (TGACGTCA) in the 5' LTR. *n*, Number of elements. Only uniquely mapping reads were considered. Statistical significance was calculated using a two-sided *t*-test, and *P* values are shown. Boxplots as in Fig. 2d. **b**, Scheme of single-copy targeted integration of different IAPLTR1a and PGK reporter constructs by recombinase-mediated cassette exchange. Bar plot shows luciferase reporter activity in neurons, indicating that the IAPLTR1a reporter is silent in WT but active in DNMT–TKO neurons. Absence of CRE reduces reporter activity by 50%. WT_PGK (*n* = 5), WT_LTR (*n* = 3), WT_LTR_∆CRE (*n* = 3), DNMT–TKO_PGK (*n* = 3), DNM–TKO_LTR (*n* = 4), DNMT–TKO_LTR_∆CRE (*n* = 4); *n*, number of biological replicates. Error bars indicate s.e.m. Statistical significance was calculated using a two-sided Wilcoxon test.

**Extended Data Fig. 8:**
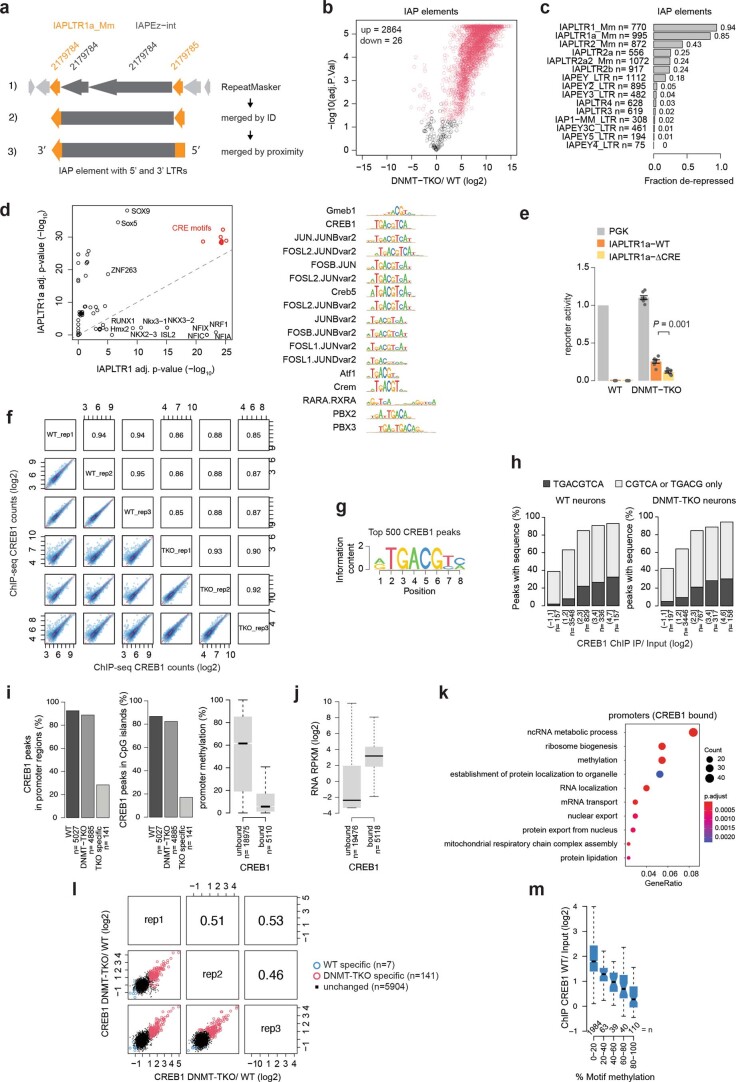
CRE is important for IAP activity and bound by methylation-sensitive CREB1. **a**, Curated RepeatMasker annotation for IAP elements. IAPLTR1a (orange) and IAPEz-int (dark grey) fragments of same ID and subfamily are merged if within 1024 bp. **b**, IAPs differentially expressed in DNMT-TKO (red, FDR < 0.05, |log2FC| >= 1) using uniquely mapping RNA reads and the curated annotation. **c**, Related IAPs are similarly de-repressed in DNMT-TKO neurons. **d**, TF motifs (black) in 5’LTR of expressed IAPLTR1/1a. Significance (Bonferroni-corrected one-sided Wilcoxon test) of expression difference (DNMT-TKO vs WT) between IAPLTR1 and 1a elements plus/minus motif. Cluster of motifs (red) reproducibly enriched in IAPLTR1 and 1a elements expressed in DNMT-TKO. Unbiased clustering (right) indicates that most resemble the CRE motif (TGACGTCA). **e**, Reporter activity in ES cells similar to Fig. 5b. IAPLTR1a reporter is silent in WT and only moderately active in DNMT-TKO ES cells. Seven biological replicates each, error bar indicates SEM. *P* = 0.0012, two-sided t-test. **f**, Reproducibility of read counts for three CREB1 ChIP-seq replicates from WT and DNMT-TKO neurons in all peak regions. Coefficients from pearson correlation. **g**, Top motif found by *de novo* motif search in the top 500 CREB1 ChIP-seq peaks shared between WT or DNMT-TKO neurons. **h**, Fraction of peaks with a CRE sequence for different bins of CREB1 enrichment in WT (left) or DNMT-TKO (right) neurons across three replicates. n = Number of peaks per enrichment bin. **i**, CREB1 peaks are located in unmethylated CpG-island promoters. n = Number of datapoints. Boxplots as in Fig. 2d. **j**, Gene expression of CREB1-bound and -unbound promoters in neurons. Boxplots as in Fig. 2d. **k**, Gene Ontology (GO) terms enriched in genes with CREB1-bound promoters (top 500). Dots represent top 10 terms with highest gene ratio (fraction of genes represented in the GO term). Dot size and color representing gene counts and adjusted *P* value (Fisher’s exact test), respectively. **l**, CREB1 binding (ChIP-seq) in DNMT-TKO versus WT cells at peak regions across cell lines indicated by red or blue circles. PCC indicated. **m**, CREB1 binding in WT neurons at all peak regions identified in WT and DNMT-TKO cells binned by motif methylation. Boxplots as in Fig. 2d.

Intracisternal A-type particle elements are characterized by 5′ and 3′ long terminal repeats (LTRs) that control the expression of the viral genes^54^ (Fig. 5a). For correct assignment of transcriptional activity to the corresponding 5′ LTR promoter region we curated the existing RepeatMasker annotation (Extended Data Fig. 8a and Methods). This revealed that almost all copies of the evolutionarily youngest types (IAPLTR1/1a) are strongly activated (Extended Data Fig. 8b,c) while divergent LTR sequences show weaker responses (Extended Data Fig. 8c).

To identify TF motifs associated with upregulation, we asked which motifs distinguish strongly from lowly upregulated IAPLTR1/1a in DNMT–TKO neurons. This revealed the cyclic AMP response element (CRE) as the top candidate (Fig. 5a, Extended Data Fig. 8d and Methods). To test the actual contribution of this motif, we generated reporter constructs driven by IAPLTR1a with or without the CRE upstream of a luciferase reporter gene (Fig. 5b) and placed them as single-copy integrants into both WT and DNMT–TKO ES cells at a defined genomic site^75^. A promoter of the *Pgk1* housekeeping gene (PGK) served as a positive control and, indeed, is equally active following insertion, while the IAPLTR is silent and efficiently repressed in WT and only weakly expressed in DNMT–TKO ES cells (Extended Data Fig. 8e). In WT cells this repression is preserved following differentiation into neurons, while in DNMT–TKO neurons the IAPLTR reporter is strongly upregulated, mimicking the activation of endogenous elements (Fig. 5b). Importantly, the CRE motif itself accounts for half of the observed transcriptional activity, suggesting that it is critical for full IAPLTR1/1a activity in the absence of DNA methylation.

### CREB1 binds unmethylated CRE in IAP elements

Although multiple TFs of the basic leucine zipper TF family can bind CRE as homo- or heterodimers^76^, the cyclic AMP (cAMP)-response element-binding protein 1 (CREB1) seemed a likely candidate at IAPLTRs because it preferentially binds CRE as a homodimer in genic and viral promoters and is furthermore ubiquitously expressed^77^.

Measurement of CREB1 genomic occupancy revealed that binding occurs at CRE or CRE half-sites (Extended Data Fig. 8f–h), which are located almost exclusively at CpG islands of unmethylated promoters of active genes (Extended Data Fig. 8i,j), many associated with general cellular functions (Extended Data Fig. 8k). Only seven sites are bound exclusively in WT while 141 are newly bound in DNMT–TKO neurons (Extended Data Fig. 8l), mainly located distal to promoters (Extended Data Fig. 8i).

**Fig. 6:**
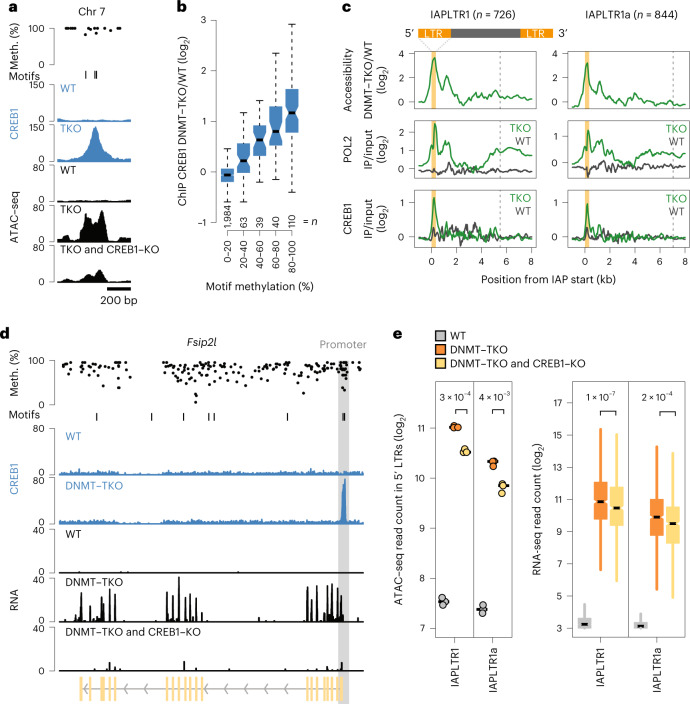
CREB1 binds in a methylation-sensitive manner to IAPLTR1/1a elements and contributes to repeat activity. **a**, Single-locus example illustrating DNA methylation-sensitive binding of CREB1 (blue tracks, ChIP–seq) in WT and DNMT–TKO (TKO) neurons. CREB1 motifs shown in the motif track. Chromatin accessibility (black tracks, ATAC–seq) for WT, DNMT–TKO and CREB1–KO in DNMT–TKO neurons. Top track indicates CpG methylation (black dots) in WT neurons. Chr7: 41118130−41118764. **b**, Changes in CREB1 binding between DNMT–TKO and WT neurons grouped according to their motif methylation in WT neurons. *n*, number of CREB1 sites indicated for each bin. Boxplots as in Fig. 2d. **c**, Changes in chromatin accessibility (top, ATAC–seq), POL2 binding (middle, ChIP–seq) and CREB1 binding (bottom, ChIP–seq) in WT and DNMT–TKO neurons at IAPLTR1/1a elements gaining expression in the absence of DNA methylation (RNA-seq, FDR < 0.05 and log_2_FC ≥ 1). Signal is centered at the start site of IAP elements. Orange bars denote average width of the 5' LTR and dashed lines average length of an entire IAP element, including the 5' and 3' LTR regions. Only uniquely mapping reads are considered. Replicates per condition are combined in each composite plot. *n*, number of elements. **d**, Genic example (*Fsip2l*) of CREB1-mediated activation following loss of DNA methylation. CpG methylation and CRE motifs are indicated; CREB1 ChIP–seq, blue tracks; gray bar indicates the promoter region methylated in WT neurons. ChrX: 48838466−48880713. **e**, ATAC–seq signal or RNA expression levels of IAPLTR1 (*n* = 746) or 1a (*n* = 884) elements in WT or DNMT–TKO neurons with (orange) or without (yellow) functional CREB1 gene at elements derepressed in the absence of DNA methylation (FDR < 0.05 and FC ≥ 2). Only reads mapping uniquely to the reference genome were considered. ATAC–seq reads counted in 5' LTRs were summed for each replicate (*n* = 3). Replicates (*n* = 2) of RNA-seq reads counted in the IAPLTR1/1a element were combined by condition. Boxplots as in Fig. 2d. Statistical significance calculated using two-sided *t*-test, and *P* values are shown.

CREB1 binding signal is inversely correlated with motif methylation in WT (Extended Data Fig. 8m) and DNMT–TKO-specific binding occurs at sites that are methylated in WT (Fig. 6a,b), arguing that CREB1 is indeed methylation sensitive in vivo, as previously predicted in vitro^25,28,78–80^.

**Extended Data Fig. 9:**
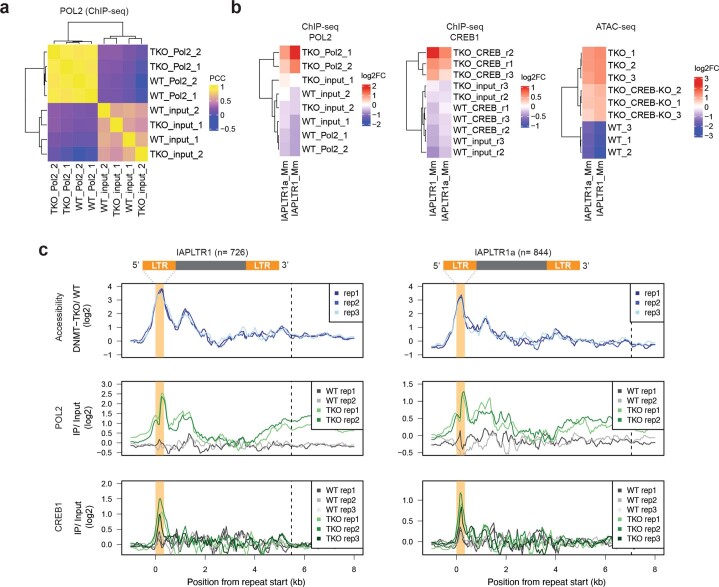
Changes in POL2 and CREB1 binding or accessibility are detectable at IAPLTR1/1a in absence of DNA methylation. **a**, Reproducibility of read counts for two independent POL2 ChIP-seq replicates from WT and DNMT-TKO neurons in promoter regions. Pearson correlation coefficients are indicated. **b**, Unsupervised clustering of changes in signal relative to mean for POL2 and CREB1 ChIP-seq or ATAC-seq samples in WT, DNMT-TKO (TKO) or CREB1-KO DNMT-TKO (TKO_CREB1-KO, only for ATAC-seq) neurons. Colors indicate pairwise Pearson’s correlation coefficients (PCC) of uniquely mapping read counts in 5’ LTRs of annotation-curated IAP elements that are de-repressed in absence of DNA methylation (RNA-seq, FDR < 0.05 and fold change > = 2). Different replicates are shown. Number of repeats 726 (IAPLTR1) or 844 (IAPLTR1a) **c**, Changes in chromatin accessibility (top tracks, ATAC-seq), POL2 binding (middle tracks, ChIP-seq) or CREB1 binding (bottom tracks, ChIP-seq) in WT and DNMT-TKO neurons at IAPLTR1/1a elements that gain expression in absence of DNA methylation (RNA-seq, FDR < 0.05 and fold change > = 2). Signal is centered at the start site of IAP elements (Methods). Orange bars depict average width of the 5’ LTR and dashed lines display the average length of an entire element including the 5’ and 3’ LTR regions. Only uniquely mapped reads are considered. Replicates are shown. n = number of elements.

Next, we asked whether CREB1 binds 5' LTR regions of IAPLTR1/1a elements in the absence of DNA methylation. To benchmark our ability to measure occupancy at repetitive sequences, we first profiled RNA polymerase II (POL2) binding in WT and DNMT–TKO neurons (Extended Data Fig. 9a). This revealed a reproducible increase in POL2 binding at 5' LTR regions of IAPLTR1/1a elements upregulated in the absence of DNA methylation (Fig. 5c and Extended Data Fig. 9b,c) and coincides with increased accessibility (Fig. 5c and Extended Data Fig. 9b,c). As expected, we did not detect POL2 binding in WT neurons at the same LTRs (Fig. 5c and Extended Data Fig. 9b,c). Quantification of CREB1 occupancy by chromatin immunoprecipitation sequencing (ChIP–seq) revealed selective and reproducible binding in the absence of DNA methylation at IAPLTR1/1a repeats (Fig. 5c and Extended Data Fig. 9b,c), indicating CREB1 binding in a methylation-sensitive manner.

### CREB1 deletion results in reduced activity at genes and IAPs

**Extended Data Fig. 10:**
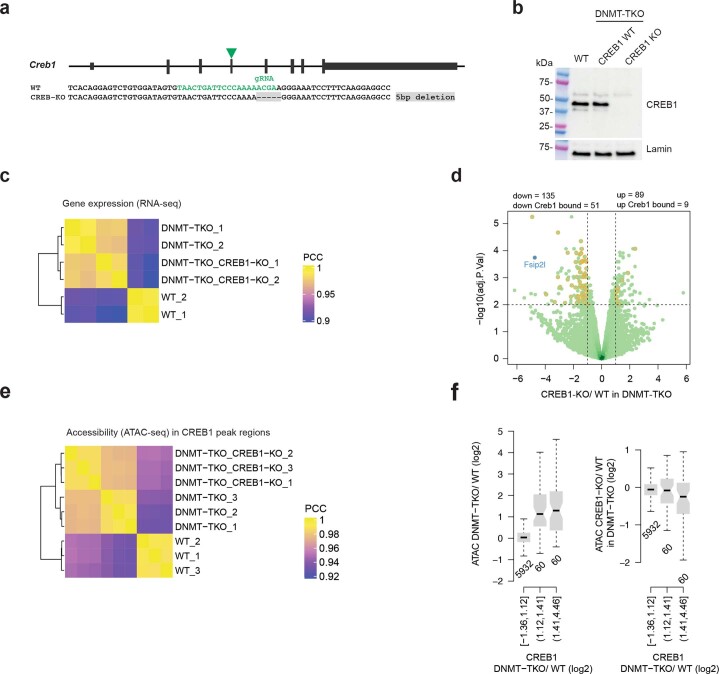
CREB1 deletion in DNMT-TKO neurons causes reduced chromatin accessibility and transcription. **a**, Scheme of *Creb1* with exons in black and CRISPR cut site indicated by green triangle. Sequence below displays a 5 bp deletion in CREB1-deleted DNMT-TKO neurons. **b**, Western blot detecting CREB1 in nuclear extracts from WT and DNMT-TKO ES cells, which is absent in CREB1-KO cells. Lamin serves as a loading control. Blot is representative of three independent experiments. **c**, Unsupervised clustering of RNA-seq signals from WT and mutant neuron cells (RPKM). PCC, Pearson’s correlation coefficient. **d**, Volcano plot showing gene expression changes between WT or CREB1-deleted DNMT-TKO neurons. Differentially expressed genes (FDR = < 0.01 and |log2FC| >= 1) indicated by dashed lines. Orange circled points are genes that are bound by CREB1 in their promoter region, as determined by ChIP-seq. *Fsip2l* is indicated by a blue dot. **e**, Unsupervised clustering of ATAC-seq samples in all CREB1 peak regions from WT, DNMT-TKO or CREB1-KO in DNMT-TKO neurons. Colors indicate pairwise Pearson’s correlation coefficients (PCC) of log-transformed normalized read counts in ATAC-seq peaks, indicating reproducibility between replicates and separation of all three genotypes. **f**, Changes in chromatin accessibility (ATAC-seq) binned by CREB1 enrichments in absence of DNA methylation. Accessibility changes between WT and DNMT-TKO neurons (left) and between WT and CREB1-deleted DNMT-TKO neurons (right). Replicate samples per condition (n = 3) are combined. Number of data points indicated. Boxplots as in Fig. 2d. Source data

To directly test CREB1 contribution to repeat activity we deleted *Creb1* in DNMT–TKO ES cells using CRISPR (Extended Data Fig. 10a,b) and generated neurons transcriptionally resembling the parent line (Extended Data Fig. 10c). Among genes bound by CREB1, the majority of responding genes were down- (*n* = 51) rather than upregulated (*n* = 9) (Extended Data Fig. 10d), in line with it being an activator^77^. Downregulated genes included *Fsip2l* (Fig. 5d), which is upregulated and bound by CREB1 at its promoter only following the removal of DNA methylation. Upregulation was reversed when *Creb1* was deleted (Fig. 5d and Extended Data Fig. 10d), providing a genic example of CREB1-mediated activation following loss of DNA methylation.

Sites that are newly bound by CREB1 and that increase in accessibility following removal of DNA methylation decrease in accessibility following *Creb1* deletion (Fig. 5a and Extended Data Fig. 10e,f). Thus CREB1 responds to genome demethylation by binding to new sites, leading to increased chromatin accessibility and transcriptional activation. Decreased accessibility is similarly evident at 5' LTRs of IAPLTR1/1a following loss of CREB1 in DNMT–TKO, accompanied by reduced transcriptional activity (Fig. 5e).

Taken together, the findings show that motif methylation of CRE abrogates binding of CREB1 to promoters of genes such as *Fsip2l* and IAP repeats. In the absence of DNA methylation, CREB1 substantially contributes to IAP upregulation. This provides a case of direct repeat repression via blockage of TF binding by motif methylation.

## Discussion

Here we asked to what extent repression of regulatory regions by DNA methylation depends on direct inhibition of binding of TFs versus indirect inhibition via sequence-independent recruitment of MBD proteins. Both stable and acute deletion of four MBD proteins with established 5mC binding in murine ES cells, differentiated neurons and a human cell line caused limited transcriptional response that appears not to be linked to methylation of regulatory sequences. This challenges a scenario where indirect repression mediated by the tested MBD proteins is essential for repression of CpG-dense methylated regulatory regions. Conversely, removal of DNA methylation results in upregulation of a group of genes controlled by otherwise methylated CpG island promoters in the tested cell states, as well as rampant transcription of endogenous retroviruses in neurons. In line with this upregulation being caused by methylation-sensitive TFs, we identify and validate new factors that are blocked from binding their motifs by DNA methylation and that activate genes and retroviruses in its absence. These results suggest that direct impediment of TF binding is a prevailing mechanism of methylation-mediated repression of regulatory regions in both human and mouse.

Importantly, these observations are compatible with other proposed functions of MBD proteins—in particular MeCP2—in gene regulation, such as impacting transcriptional elongation by methyl-CA binding^65,81,82^, alternative splicing^83,84^, microRNA processing^85^ or protecting CA repeats from nucleosome invasion^86^. While MBD proteins can have a repressive function—in particular when recruited to certain sites or at transfected reporter plasmids^17–19,87–90^—our experiments argue against functional redundancy between the four tested MBD proteins as a reason for the absence of more severe transcriptional phenotypes, as hypothesized in previous loss-of-function studies of selected MBD proteins^19,36^. It remains conceivable that the MBD proteins we tested participate in stabilizing aspects of transcriptional repression^91,92^ in a way that is redundant in the cell systems we employed, yet relevant in vivo in different contexts. It remains possible that other, currently uncharacterized, sequence-agnostic methyl-CpG binding proteins exist and are able to mediate indirect repression. TAM domain proteins^7,8^ seem unlikely candidates because they do not bind methylated DNA^10,11^ and show only weak homology in the MBD domain. The plant-specific MBD5 and MBD6 are readers of methylated DNA and mediate transcriptional repression at a subset of genes and repeats^93^ via the recruitment of chaperone activity, yet are unrelated and nonhomologous.

In contrast to the mild phenotype of MBD deletions, we did observe that methylation of CpGs within specific motifs interferes with TF binding. Removal of DNA methylation increases chromatin accessibility, TF binding and transcription, both genome wide and in reporter assays. In addition to factors shown to be methylation sensitive in cells at their canonical motif (NRF1, BANP, CREB1), we report ONECUT1 to be methylation sensitive at only one specific CpG-containing motif variant, but not the CpG-free canonical motif. This agrees with previous in vitro observations in a SELEX-based screen^28^ and defines the actual contributions of these variants to the ONECUT1 binding landscape in the cellular context.

Structural data of CREB1 (ref. ^94^) and ONECUT1 (ref. ^95^) in complexes with unmethylated DNA show that both proteins interact with the major groove where the methyl group of the cytosine is positioned^22^, causing groove widening^96^. CREB1 does not bind if the central cytosine is replaced by a thymidine, which structurally resembles methyl-cytosine^97^. Of note, methylation can also change the DNA shape at neighboring base pairs, thus affecting binding for motifs that do not contain central CpGs^29,96^. It is an intriguing possibility that methylation-restricted binding at select TF motifs can function to mediate TF hierarchies^30^ or specifically regulate different motif variants in a cell type-specific manner, thus expanding the gene regulatory toolkit at a subset of sites. Although comparison with ancestral genomes reveals ongoing depletion of CpG-containing TF motifs^98^, a large fraction of promoters is rich in CpGs and these are indeed efficiently silenced by DNA methylation. We speculate that this is due to a combination of inhibition of methylation-sensitive TFs with complex motifs, but also to CXXC-domain-containing proteins that bind unmethylated CG dinucleotides and have been linked to activation^99^.

It is unclear whether aberrant gene expression^43^ or repeat activation^44^ causes cellular death in differentiated cells in the absence of DNA methylation^96^. While both processes have been linked to mitotic catastrophe in dividing cells^38^, our methylation-devoid neurons are postmitotic for several days before cell death, suggesting alternative scenarios in nondividing cells. Rampant repeat activation is the key feature that distinguishes these neurons, which potentially induces cell death by sheer transcriptional load, activation of the interferon pathway^100^ or insertion of active endogenous retroviruses (ERVs) into genes or promoter regions, thereby producing mutations or high levels of chimeric transcripts^101^.

Release of direct inhibition of methylation-sensitive TFs such as CREB1 contributes to repeat activation in differentiated cells. DNA methylation-independent pathways repress repeats in vertebrates during periods of global low methylation that occur in the germline as part of epigenome resetting^41^. Transcription and transposition in the germline is critical for genomic expansion of ERVs and thus for their evolutionary ‘success’^102^, whereas their activity in somatic cells would only reduce the fitness of the host. Transcriptional control by methylation-sensitive TFs could benefit the expansion of ERV by being compatible with expression in hypomethylated states in the germline while ensuring repression in somatic cells. It enables exploitation of an ubiquitously expressed activator such as CREB1 and might contribute to the fact that IAP elements are among the most active TEs in the mouse genome^103^.

The larger family of ERVK elements to which IAP elements belong includes human counterparts, the HERVK LTR retrotransposons, of which HERVK(HML-2) appears to replicate in the human population^102^. Interestingly, several human LTR retrotransposons contain CRE motifs and CREB or ATF/AP-1 factors have been implicated in driving the expression of human ERVs, human T cell leukemia virus type 1 and human immunodeficiency virus^104–106^. CRE methylation has furthermore been linked to promoter silencing of the Epstein–Barr virus genome^79^.

Taken together, our findings provide insights into transcriptional repression through DNA methylation of CpG-rich regulatory regions that drive genes and repeats, and favor a model of direct inhibition of TF binding as the prevailing molecular mechanism. This finding is in line with a model where genome-wide DNA methylation evolved as an efficient means to repress repetitive elements in somatic cells and was subsequently co-opted to other regulatory regions, resulting in an epigenetic marking system that remains essential at the cellular level in somatic cells.

## Methods

### Cell culture

HA36 mouse ES cells (mixed 129-C57Bl/6 strain, no commercial source available) were maintained in DMEM (Invitrogen), supplemented with 15% fetal calf serum (Invitrogen), 1× GlutaMax (Thermo Scientific), 1× nonessential amino acids (Gibco), 0.001% beta-mercaptoethanol (Sigma) and leukemia inhibitory factor (LIF; produced in house). All experiments were performed with cells grown for several passages on plates coated with 0.2% gelatin (Sigma).

HEK293 cells (obtained from ATCC, no. CRL-1573) were cultured in DMEM (Invitrogen), supplemented with 10% fetal calf serum (Invitrogen) and 2 mM L-Glutamine (Thermo Scientific).

### Cell line generation

#### *Ngn2* cassette integration

Mouse ES cells (HA36, 4 × 10^6^ cells) were electroporated (mouse ES cell Nucleofector Kit, no. VPH-1001, Amaxa biosystems) in 100-µl volumes containing 95 µl of Nucleofector solution, a Piggybac plasmid containing a cassette with doxycycline-inducible *Ngn2* (3.8 µg) and Dual helper construct (0.7 µg). Electroporated cells were cultivated in 2i/LIF maintenance medium (G-MEM BHK-21 medium containing 10% KnockOut serum, 1 mM sodium pyruvate, 1× nonessential amino acids, 0.1 mM B-mercaptoethanol, LIF, 1 µM PD0325901 and 3 µM CHIR99021 inhibitors) on gelatin-coated dishes. After 2 days, G418 (300 µg ml^–1^) was added to the cells for 2 weeks to select those that integrated the Piggybac cassette. Individual clones were then tested for *Ngn2* expression and neuronal differentiation.

#### MBD–TKO and MBD–QKO mouse ES cells

MBD double-knockout ES cells (MBD–DKO) were generated by cotransfecting (Lipofectamine 3000, Thermo Fisher Scientific) HA36 cells containing an integrated *Ngn2* cassette with two vectors, each encoding CRISPR–Cas9 and a gRNA against either *Mbd2* or *Mecp2*. Two distinct gRNAs were used to target each gene, to generate two biological replicates. Puromycin-resistant clones were genotyped for frameshift mutations by PCR, expanded and MBD–DKO clones validated by immunoblot. To generate MBD–QKO, the same process was repeated using MBD–DKO cells with the addition of gRNAs targeting *Mbd1* and *Mbd4*. The MBD–TKO cell line lacking *Mbd1*/*Mbd2*/*Mecp2* was generated by deletion of *Mbd1* from MBD–DKO with the second set of gRNAs. Details of all gRNAs used for generation of mouse ES cells can be found in Supplementary Table 1.

#### MBD–QKO HEK293 cells

HEK293 cells were cotransfected with plasmids encoding either CRISPR–Cas9 or the gRNA sequence with a red fluorescence protein. In a first step, *MBD2* and *MECP2* were targeted simultaneously and thus RFP^+^ HEK293 cells were sorted (BD FACS Aria III) into 96 wells and genotyped. Double-knockout clones carrying a frameshift mutation were expanded and validated by allele sequencing and immunoblot. In a second and third step, this process was repeated twice targeting *MBD1* and *MBD4* successively to delete all four MBD proteins. gRNAs used for HEK293 can be found in Supplementary Table 2.

#### DNMT–TKO mouse ES cells

The three DNMTs—*Dnmt1*, *Dnmt3a* and *Dnmt3b*—were deleted in HA36 ES cells with the integrated *Ngn2* cassette by CRISPR–Cas9 gene editing as previously described^30^, to generate a DNMT–TKO line without DNA methylation. *Dnmt* genes of all six alleles were sequenced and residual methylation levels measured by Zymo Research, using high-pressure liquid chromatography coupled to mass spectrometry to confirm successful targeting.

#### CREB1–KO in DNMT–TKO mouse ES cells

Mouse HA36 DNMT–TKO ES cells generated as described above were cotransfected (Lipofectamine 3000, Thermo Fisher Scientific) with one vector encoding CRISPR–Cas9 and a gRNA (TAACTGATTCCCAAAAACGA) against *Creb1*, in addition to a puromycin selection marker. Puromycin-resistant clones were genotyped for frameshift mutation by PCR, expanded and validated by immunoblot.

All generated cell lines are available upon request.

### Antibodies

Antibodies used in this study for immunoblot and ChIP–seq experiments are listed in Supplementary Table 3 (mouse) and Supplementary Table 4 (human).

### siRNA-mediated knockdown and RNA-seq

For knockdown of *Setdb1*, 50,000 ES cells per well were seeded in a six-well plate and simultaneously transfected with either 7.5 µl of 20 µM *Setdb1* siRNA (Dharmacon, no. M-040815-01-0005) or Allstars negative control from GeneSolution siRNA (Qiagen, no. 1027281) using Lipofectamine RNAiMAX (Invitrogen, no. 13778-075). Medium was exchanged after 24 h and transfection repeated after 48 h. Duplicates for each condition were harvested after 72 h, RNA isolated with Direct-zol RNA Microprep (Zymo research, no. R2061) and converted to complementary DNA using the PrimeScript RT reagent Kit (Takara, no. RR047A). Expression levels of genes or repeats were measured with quantitative PCR primers against *Gapdh*^108^, *Setdb1* (ref. ^108^) or IAP-gag^74^. For knockdown of *Mbd4*, 200,000 ES cells per well were seeded in a six-well plate and simultaneously transfected with 7.5 µl of *Mbd4* siRNA (20 µM) from GeneSolution siRNA (Qiagen, no. 1027416). After 24 h. cells were harvested for immunoblot or RNA-seq.

### 5-Aza treatment of HEK293 cells

Wild-type or MBD–QKO HEK293 cells (150,000 seeded the day before in a well of a six-well plate) were treated with either 1 μM 5-Aza-2′-deoxycytidine (no. A3656-10MG, Sigma) or DMSO in triplicate. The next day, the medium was replaced with fresh Aza or DMSO. After 72 h cells were harvested for RNA isolation.

### Neuronal differentiation

For HA36 cells containing the pTRE-*Ngn2* construct, differentiation was carried out by inducing expression of NGN2 with doxycycline as previously described^63^, with the following modifications. Cells were plated on poly-d-lysine/laminin-coated plates and treated with DMEM/F12 and Glutamax (LifeTech, no. 31331-028) containing 1× B27 without vitamin A (LifeTech, no. 12587-010), 1× N2 supplement (LifeTech, no. 17502-048), 10 ng ml^–1^ human epidermal growth factor (LifeTech, no. PHG0315), 10 ng ml^–1^ human fibroblast growth factor (LifeTech, no. CTP0261) and 1 μg ml^–1^ doxycycline (Sigma, no. D989) for 3 days with no medium change. At day 3 after doxycycline induction, medium was changed to Neurobasal-Medium (LifeTech, no. 21103-049) supplemented with 1× B27 and Vitamin A (LifeTech, no. 17504-044), 1× N2 (LifeTech, no. 17502-048), 10 ng ml^–1^ brain-derived neurotrophic factor (PeproTech, no. 450-02), 10 ng ml^–1^ glial cell line-derived neurotrophic factor (PeproTech, no. 450-10) and 10 ng ml^–1^ NT-3 (PeproTech, no. 450-03). Every other day, half of the medium was replaced with fresh. RNA-seq, ChIP–seq and ATAC–seq were performed 8 days after doxycycline induction.

### Quantification of cell viability

A mix of nuclear and cell death markers (1 µl of Hoechst, 8 µl of propidium iodide and 10 µl of AnnexinV in 125 µl of AnnexinV binding buffer (Thermo Fisher, no. V13242)) were added to neuronal cell culture in six-well plates at days 8 and 10. After 15 min of incubation at 37 °C, images were acquired with a ZOE Fluorescent Cell imager (Bio-Rad, no. 145-0031) and analyzed using ImageJ^109^. In brief, nuclei were segmented based on Hoechst signal and the background-subtracted AnnexinV-PI signal was measured in each segmented cell. Between the two cell populations separated based on viability markers, cells without AnnexinV-PI enrichment were counted as healthy.

### Recombinase-mediated cassette exchange

For targeted insertion, the IAPLTR1a_Mm consensus sequence (downloaded from repbase^110^) or *Pgk1* promoter region (chrX:106186728-106187231, GRCm38/mm10 genome) was cloned into a plasmid containing a multiple cloning site flanked by two inverted L1 *Lox* sites. Recombinase-mediated cassette exchange was performed in HA36 mouse ES cells as previously described^75^.

### Luciferase assay

Luciferase activity of ES cells or derived neurons (8 days after induction) carrying a IAPLTR1a or PGK luciferase reporter was measured with the Luciferase Assay System (Promega, no. E1500) according to the manufacturer’s instructions. Normalization was carried out by protein concentration of lysed ES cells or neurons in 1× lysis buffer with Protein Assay (Bio-Rad, no. 500006). Luminescence was measured using a luminometer (Berthold Technologies, Centro XS3 LB 960).

### RNA-seq

RNA was isolated from pellets of either (1) ES or HEK293 cells with the RNeasy mini kit (Qiagen, no. 74104) using on-column DNA digestion or (2) neurons (8 days after doxycycline induction) with Direct-zol RNA Microprep (Zymo research, no. R2061) with on-column DNA digestion. Sequencing libraries were prepared from purified RNA for a minimum of two biological replicates per condition using TruSeq Stranded Total RNA Library Prep Gold (Illumina, no. 20020599). ES cell libraries were single-end sequenced on a HiSeq 2500 platform with 50 cycles. lllumina RTA 1.18.64 (HiSeq 2500) and bcl2fastq2 v.2.17 were used for base calling and demultiplexing.

HEK293 or neuron libraries were sequenced on an Illumina NextSeq platform with paired-end reads of 2 × 38 or 2 × 75 base pairs (bp), respectively. Illumina RTA 2.4.1 (NextSeq 500) and bcl2fastq2 v.2.17 were used for base calling and demultiplexing.

RNA of *Mbd4* or control siRNA-treated MBD–TKO ES cells (in triplicate) was isolated using Direct-zol RNA Microprep (Zymo research, no. R2061). Sequencing libraries were prepared using TruSeq Stranded Total RNA Library Prep Gold (Illumina, no. 20020599) and paired-end sequenced on a NovaSeq 6000 platform with 2 × 56 cycles. lllumina RTA 3.4.5 (NovaSeq 6000) and bcl2fastq2 v.2.20 were used for base calling and demultiplexing.

### ChIP–seq

ChIP was carried out as previously described^111^ with the following modifications. (1) Chromatin was sonicated for 20 cycles of 30 s using a Diagenode Bioruptor Pico, with 30-s breaks between cycles; (2) Dynabeads protein A (Invitrogen, no. 10008D) was used; and (3) DNA was purified using AMPure XP beads. Immunoprecipitated and input DNA were submitted for library preparation (NEBNext Ultra DNA Library Prep Kit, Illumina, no. E7370). In the library preparation protocol, input samples (200 ng) were amplified using six PCR cycles and immunoprecipitation samples using 12 cycles. Libraries were paired-end sequenced for 150 cycles (2 × 75 bp) on the Illumina NextSeq 500 platform. Illumina RTA 2.4.1 (NextSeq 500) and bcl2fastq2 v.2.17 were used for base calling and demultiplexing.

### ATAC–seq

ATAC–seq was performed according to the protocol previously described for Omni-ATAC^112^ for both ES and neuronal cells. Briefly, 50,000 cells were washed with cold PBS and resuspended in lysis buffer to extract nuclei, which were then cold-centrifuged at 500*g* for 10 min. Nuclear pellets were incubated with transposition reaction buffer for 30 min at 37 °C. DNA was purified using the PCR Purification Kit (Qiagen). Eluted transposed DNA was amplified with 11–12 cycles of PCR using Q5 High-Fidelity Polymerase (NEB). Libraries were sequenced paired-end with 76 cycles (2 × 38 bp) on the Illumina NextSeq platform. All ATAC–seq experiments were performed in at least two independent biological replicates per condition. Illumina RTA 2.4.1 (NextSeq 500) and bcl2fastq2 v.2.17 were used for base calling and demultiplexing.

### Whole-genome bisulfite sequencing

Nuclei of day 8 neurons were isolated as described by Grand et al.^47^ and sorted by flow cytometry (BD FACS Aria III). Genomic DNA was isolated (QIAamp DNA Micro Kit, no. 56304) from mouse ES cells or sorted neuronal nuclei and 1 µg was fragmented (Covaris S220) to an average size of ~300 bp. Libraries were prepared according to the manufacturer’s instructions. Adapter ligation was performed using the NEBNext Ultra II DNA Library Prep Kit (no. E7645L) with methylated adapters (NEBNext, no. E7535S), bisulfite treated (EZ DNA Methylation-Gold Kit; Zymo, no. D5006) and indexed (NEBNext Multiplex Oligos for Illumina) using 11 cycles in the PCR reaction (KAPA HiFi HotStart Uracil+ ReadyMix; Roche, no. 07959052001). Libraries were paired-end sequenced on a NovaSeq 6000 platform with 2 × 100 cycles. lllumina RTA 3.4.5 (NovaSeq 6000) and bcl2fastq2 v.2.20 were used for base calling and demultiplexing, with one sample per genotype. WT neuron experiments were performed in duplicate (individual differentiation experiments) and sequenced to half the coverage compared with the other samples.

HEK293 genomic DNA was isolated with a QIAamp DNA mini kit (Qiagen, no. 51306) and fragmented with Covaris S220, with 500 ng of fragmented DNA then used for library preparation (NEBNext Ultra DNA Library Prep Kit; NEB, no. E7370) with methylated adapters (NEBNext; NEB, no. E7535S) and bisulfite treated with EZ DNA methylation-lightning Kit (Zymo Research, no. D5046). Final PCR amplification was performed using a KAPA HiFi HotStart Uracil+ ReadyMix PCR Kit (Roche, no. 07959052001) with 12 cycles of amplification. One sample was prepared per genotype.

The resulting libraries were sequenced on an Illumina NextSeq platform (75 cycles, single-end). Illumina RTA 2.4.1 and bcl2fastq2 v.2.17 were used for base calling and demultiplexing.

### Statistics and reproducibility

No statistical method was used to predetermine sample size. No data were excluded from the analyses. The experiments were not randomized and the investigators were not blinded to allocation during experiments and outcome assessment. All statistical tests and number of observations are stated in figure panels or legends. Resulting *P* values are two-sided, with exceptions stated in individual figure legends.

In all boxplots, black lines correspond to median, boxes to first and third quartiles and whiskers to 1.5 times the interquartile range (IQR). Notches, if indicated, extend to ±1.58 × (IQR/sqrt(*n*)). Whiskers correspond to the maximum and minimum values of the distribution after removal of outliers, in which outliers were defined as >1.5 × IQR away from the box. Pearson correlation coefficients were calculated using the R function cor, with default parameters.

### Reporting summary

Further information on research design is available in the Nature Portfolio Reporting Summary linked to this article.

## Online content

Any methods, additional references, Nature Portfolio reporting summaries, source data, extended data, supplementary information, acknowledgements, peer review information; details of author contributions and competing interests; and statements of data and code availability are available at 10.1038/s41588-022-01241-6.

## Supplementary information

Supplementary Information Supplementary Methods

Reporting Summary

Supplementary Table gRNA and antibody information.

## Data Availability

All datasets that were generated in this study were deposited at Gene Expression Omnibus (GEO, https://www.ncbi.nlm.nih.gov/geo/) under accession no. GSE184470. The following public RNA-seq datasets were obtained from GEO: P5 mouse cortex *cUhrf1* KO (GSM2241736/GSM2241739/GSM2241740) and matching heterozygote (GSM2241735/GSM2241737)^61^; ES *cSetdb1 cDnmt1* KO (GSM2059172/GSM2059173) and matching WT (GSM2059171)^74^; and E8.5 whole embryos *Dnmt1*-KO (GSM3752651/52/53) and matching WT (GSM3752646/GSM3752647/GSM3752648)^68^. For the analysis of non-CpG methylation, CA methylation levels of chromosome 1 from Lister et al.^66^ were downloaded from GEO (GSE47966). The Jaspar2018 (ref. ^113^) motif database used in this study can be accessed online (https://jaspar2018.genereg.net/)^114–128^. The RepeatMasker (http://www.repeatmasker.org) annotation used in this study was downloaded from the UCSC genome annotation database for the December 2011 (GRCm38/mm10) assembly of the mouse genome (ftp://hgdownload.cse.ucsc.edu/goldenPath/mm10/database/rmskOutBaseline.txt.gz). Source data are provided with this paper.

## Source data

Source Data Fig. 1 Unprocessed immunoblots.
Source Data Extended Data Fig. 1 Unprocessed immunoblots.
Source Data Extended Data Fig. 2 Unprocessed immunoblots.
Source Data Extended Data Fig. 10 Unprocessed immunoblots.
