# Supplementary material for: Evidence that direct inhibition of transcription factor binding is the prevailing mode of gene and repeat repression by DNA methylation
Source: Nat Genet. 2022 Dec 5;54(12):1895–906. doi: 10.1038/s41588-022-01241-6 (PMC9729108; doi:10.1038/s41588-022-01241-6)
Supplement: Supplementary file 1 — Supplementary Methods [file 41588_2022_1241_MOESM1_ESM.pdf]

nature genetics

Article https://doi.org/10.1038/s41588-022-01241-6

Evidence that direct inhibition of transcription factor binding is the prevailing mode of gene and repeat repression by DNA methylation

In the format provided by the authors and unedited

Supplementary Methods

Computational analysis

Annotations

Three sets of mouse promoters were defined as follows: 1. General_promoters: 500 bp down- and 1000 bp upstream of the TSS, using gene annotation from TxDb.Mmusculus.UCSC.mm10.knownGene (v3.10.0) where one promoter was randomly chosen per gene. 2. Pol2_wt_promoters: same as general promoters but selecting the promoter with the strongest POL2 ChIP enrichment (IP vs Input, see below) in WT neurons per gene. 3. Pol2_tko_promoters: same as Pol2_wt_promoters but using POL2 ChIP enrichments in DNMT-TKO neurons. The RepeatMasker (http://www.repeatmasker.org) annotation was downloaded from the UCSC genome annotation database for the Dec. 2011 (GRCm38/mm10) assembly of the mouse genome (ftp://hgdownload.cse.ucsc.edu/goldenPath/mm10/database/rmskOutBaseline.txt.gz).

To curate the RepeatMasker annotation of IAP proviruses, genomic ranges annotated as IAPs were grouped based on the “repName” and “ID” columns that contained the word “IAP” (Extended Data Fig. 8a). The “ID” column describes related ranges and is provided by RepeatMasker. In a group, the start position of the first and the end position of the last range were selected, and a new range was generated (henceforth called “ID fragment”). Subsequently, ID fragments of the same subfamily were combined when located closer than 1024 bp, with the assumption that fragments located in proximity belong to the same provirus. This curation resulted in an annotation that describes the entire length of a provirus (IAP element) including the associated five prime (5’ LTR) and/or three prime LTR (3’ LTR).

Human promoters were defined by randomly selecting one TSS per gene using annotation from TxDb.Hsapiens.UCSC.hg19.knownGene (v3.2.2) and expanding the region 500 bp down- and 1000 bp upstream.

RNA-seq analysis in genes and repeats

For gene and repeat expression analysis, reads were aligned to the mm10 or hg19 genome using QuasR[114] (v1.32.0) using the aligner Hisat2 with parameters set to splicedAlignment=T, aligner= "Rhisat2", and maxHits=1 to select only uniquely mapping reads or maxHits=100 for selecting multi-mapping reads[115] (reads mapping to up to 100 locations are randomly assigned to one of these) To quantify gene and repeat expression levels, alignments overlapping the opposite strand (first read for paired-end data) of any exon of a gene (using uniquely mapping alignments) accessed via the Bioconductor[116] package TxDb.Mmusculus.UCSC.mm10.knownGene (v3.10.0) or TxDb.Hsapiens.UCSC.hg19.knownGene (v3.2.2) or repeats as defined by RepeatMasker were counted using the *qCount* function from the QuasR package with default parameters. Counts in repeats were collapsed by repeat subfamily ("repName"). Differential expression analysis for genes and repeats from the RepeatMasker annotation was performed using TMM normalization[117] combined with significance calculations using voom from the limma R package[118,119] with default settings as described in the limma vignette. Weakly or non-detected genes or repeats were removed using *filterByExpr*[120] with default settings. To determine the ratio of repeat vs genic expression, for each sample the total number of repeat counts (excluding gene-overlapping regions) were divided by the total number of genic counts (excluding repeat overlapping regions).

Over-representation of gene ontology (GO) categories was determined in selected gene sets using clusterProfiler v4.0.5[121] with default settings, using all genes as background. The redundancy of enriched GO terms was removed using *simplify* from clusterProfiler with default settings. In the GO plots, dots represent top terms with highest gene ratio (fraction of genes represented in the given GO term). Dot size and color representing gene counts and adjusted *P* value (Fisher's exact test), respectively.

For the direct comparison to published datasets, all of our own and public total RNA-seq samples were trimmed to 50 bp (to account for varying read lengths) and aligned to the mm10 genome using Hisat2 in QuasR with splicedAlignment=T, aligner= "Rhisat2" considering unique (maxHits=1) or multi-mapping reads (maxHits = 100),

using only the first read in the case of paired-end datasets The number of alignments in repeats were determined using *qCount* with default settings (except orientation = “opposite” for all samples generated with stranded protocols). When counting in multi-mapping mode, read counts were collapsed to subfamily (“repName”) level for further analysis. In all cases, counts were normalized to the smallest total number of genic alignments (of all samples) and log2 transformed after adding a pseudo-count of eight. Finally, read counts were averaged over replicates.

ChIP-seq analysis

Paired-end (2 x 75bp) ChIP-seq reads were aligned to mm10 using *qAlign* from the QuasR package (using Rbowtie 1.26.0[114] / bowtie 1.2.2[122]) with default parameters, only reporting alignments for reads with a unique match to the genome. For TF ChIP–seq data, peaks were called using macs2 v.2.2.6[123] on the bam files of the IP samples using *callpeak* with parameters *-t IP.bam --f BAMPE -g mm -q 0.05*.

Aligned fragments in peaks were counted using *qCount,* whereby the fragment midpoints were used to define fragments overlapping with a peak (shift=”halfInsert” and useRead=”first”). Peaks were defined as peak summits resized to 201 or 301 bp for ONECUT1 or CREB1, respectively, and merged across WT and DNMT-TKO samples, whereby all overlapping peaks where combined into single peaks containing all nucleotides of the individual overlapping regions. Counts were normalized to the smallest library size, log2-transformed after adding a pseudo-count of eight and finally quantile normalized using the function *normalizeQuantiles* from the limma package (v3.48.3)[118,119]. Log2 changes in binding between samples or conditions were calculated as the difference in log2 counts as defined above.
CREB1 IP replicates two and three (both WT and DNMT-TKO) were paired with their matching input samples, whereas the first IP replicates were matched with the second input replicates, as no matching input samples had been generated. Only peaks with an enrichment (IP vs input) of at least two-fold in at least two replicates were retained for further analysis. For each TF, a binding motif was identified using the top 500 peaks ordered by enrichment using the function *findMotifsGenome.pl* from HOMER[124] (v.4.11, with parameters *-len 8,10,12,14 -size given -noknown*. The best (first) motif identified is depicted in Extended Data Fig. 8g. For GO enrichment analysis (as

described above) CREB1 ChIP-seq reads were counted in Pol2_wt_promoters (see Annotations). Promoter enrichments in WT neurons were calculated by subtracting the input from the IP samples (after log2-transformation as described above) and averaging log2 counts over all replicates. Finally, the top 500 enriched WT promoters were used for GO enrichment analysis.
POL2 ChIP-seq counts were normalized and log2-transformed in the same way as the TF ChIP data-sets, but not quantile-normalized.

ATAC-seq analysis

ATAC-seq reads were trimmed using cutadapt v2.5, with parameters -a CTGTCTCTTATACACATCT -A CTGTCTCTTATACACATCT -m 5 –overlap=1 and then mapped to mm10 using QuasR with default parameters, allowing only for uniquely mapping reads. Mitochondrial reads were subsequently removed using samtools[125] (v.1.9). Peaks of individual replicates were called on bam files using macs2 v.2.2.6 using *callpeak* with parameters *-t IP.bam -f BAMPE -g mm -q 0.05*. Mapped alignments were counted in either all peak regions occurring in at least two replicates per condition using the *qCount* function from the QuasR package with default parameters. Counts were normalized to the sample with the smallest number of alignments in Pol2_tko_promoters and log2-transformed after adding a pseudo-count of eight. Log2 fold-changes were defined as the difference in log2-transformed counts.
Significance analysis of differential accessibility was performed using TMM normalization combined with voom (using quantile-normalization (normalize="quantile")) from the limma R package.

ATAC–seq metaprofiles centered around NRF1 (from JASPAR2018[113], MA0506.1) or BANP[47] motifs were generated using the *qProfile* function from the QuasR package, which, with default parameters, counts the total number of 5' ends of mapped reads aligning to each position relative to the corresponding anchor point (here NRF1 and BANP motifs). These counts were scaled down to the smallest library size and then smoothed with a running mean of 51 bp. For visualization, metaprofiles were rescaled such that the largest value in any shown sample was one.

For Extended data Fig 4f, each ATAC-seq peak was assigned to its closest TSS, using only TSS of expressed genes as defined by the *filterByExpr* function of edgeR with default settings.

To search for TF motifs enriched in DNMT-TKO specific ATAC-seq peak regions (FDR < 0.01 and log2FC > 3), the small number of regions overlapping with CpG islands (4 %) were removed as CpG islands have a very different nucleotide composition and can bias motif finding analysis. TF motifs were searched using *findMotifsGenome.pl* from HOMER with parameters *-size given -mknown* using the JASPAR2018 motif database.

RNA-seq analysis in IAPs

To determine expression levels of IAP elements, uniquely mapping RNA reads were counted in curated IAP elements (see Annotations) using the QuasR function *qCount* (with orientation = "opposite" and useRead = "first"). Differential expression analysis for IAP elements was performed using voom from the limma R package with default settings. As input to voom, the library size of each sample was set to the sum of reads mapping to genes and the normalization factor to the ratio of the minimal sum of reads mapping to genes across all samples divided by the sum of reads mapping to genes of each respective sample. This normalization was chosen as it only assumes that there is no global change in RNA expression at genes, while it does not, unlike a normalization to library size, assume no global change in RNA expression at genes and repeats taken together. Weakly or nondetected IAP elements were removed using the *filterByExpr* function.
To compare RNA expression levels of upregulated (FDR < 0.05 and fold change > 2) IAP elements between DNMT-TKO neurons and WT or knock-out CREB1 neurons **(Fig. 5g)**, RNA counts were normalized to the smallest total count of genic alignments. Subsequently, counts were log2-transformed after adding a pseudo-count of eight and log2 changes calculated as the difference in the log2-transformed values.

WGBS-seq analysis

Human (single-end) or mouse (paired-end) WGBS-seq reads were aligned to genomes of either hg18 (for comparison to Lister et al.[66]), hg19 or mm10, respectively,

using *qAlign* from the QuasR package with default parameters (with bisulfite = ”dir”). Total and methylated counts for Cs in the CpG context (genome-wide) and in the CpA context (only on chr1) were calculated using the qMeth function from QuasR. To retrieve CpG methylation levels of 1 kb windows, promoters or ATAC-seq peaks, the sum of methylated C counts were divided by the total number of counts for Cs in a given region. For comparison with CA methylation levels, the mouse data (chromosome 1) from Lister et al.[66] was lifted from mm9 to mm10 using the R package *rtracklayer*[126].

Motif identification in IAPs

To identify TF motifs important for IAP activity, IAPLTR1 or IAPLTR1a elements were split into two groups based on the presence or absence of any TF motif in the JASPAR 2018 database, whereby a motif match was defined as a sequence element with log-odds score >= 10 (over a uniform background) in the 5’ LTR. For any TF motif, weakly expressed or non- detected IAP elements were removed from both groups using the *filterByExpr* function (as described above) and only motifs that occurred in more than ten and not in all 5’ LTR instances were considered for further analysis. For each motif, RNA expression fold changes (DNMT-TKO vs WT neurons) for both groups were calculated, and the significance of the expression difference determined by a Wilcoxon test (one-sided) and subsequent multiple-testing correction (Bonferroni).

ChIP-seq and ATAC-seq analysis in IAPs

To investigate CREB1 and POL2 binding in IAP elements, uniquely mapped ChIP-seq fragments were counted using *qCount* (with shift = "halfInsert", useRead = "first") in all curated IAP 5’ LTRs that are significantly upregulated in DNMT-TKO vs WT neurons (RNA-seq, FDR < 0.05 and fold change > 2). Fragment midpoints were used to define overlaps with each element. Counts from each sample were summed by repeat subfamily (“repName”) and normalized to the smallest library. Subsequently counts were log2-transformed after adding a pseudo-count of 8.
To determine chromatin accessibility levels, the same analysis was repeated by counting ATAC-seq reads with qCount (default settings) but normalized to the sample with the smallest number of alignments in Pol2_tko_promoters (see Annotations).

ChIP-seq or ATAC-seq meta-profiles were generated with the QuasR function *qProfile* as follows: uniquely mapped reads were counted in -1000 nt and +8000 nt from the start position of curated IAP elements (expressed in DNMT-TKO neurons, FDR < 0.05 and log2FC > 1) and normalized as above. For ChIP-seq, fragment midpoints were counted, whereas for ATAC-seq, the 5' ends of each read were counted as they correspond to the transposase insertion sites. Profiles were then smoothed with a running sum of 201 bp to account for noise resulting from low read numbers due to an impaired mappability rate in repetitive regions. Finally, counts for each sample were log2-transformed after adding a pseudo-count of eight. For CREB1, as in the analysis outside of IAPs, IP replicates two and three (both WT and DNMT-TKO) were paired with their matching input samples, whereas the first IP replicates were matched with the second input replicates, as no matching input samples had been generated. Log2 enrichments over input were calculated by subtracting the log2-transformed counts of the input from the IPs.

Kmer enrichments at distal ATAC-seq peaks

ATAC-seq peaks outside of CpG islands were resized to 401 nucleotides around their midpoints (in order to avoid larger contributions to hexamer counts from longer peaks) and split into bins according to their changes in ATAC-seq signal. For each bin separately we then performed the following steps: The number of occurrences of each hexamer in each peak sequence was determined. To avoid a strong influence of very repetitive sequences, we performed zoops (zero-or-one-occurrence) counting, i.e., a hexamer that occurred multiple times in a peak sequence was counted only once. We then summed all the hexamer counts over all peak sequences resulting in a set of foreground hexamer frequencies. As a background model, we estimated expected hexamer frequencies using a second-order Markov model. To be consistent with zoops counting, we fitted the Markov model not on the original peak sequences, but on an artificial set of hexamer sequences that contained each hexamer as many times as its foreground frequency. Finally, we calculated, for each hexamer, its log2 enrichment as the difference in log2 counts between foreground and background after adding a pseudo-count of 8.

All single locus plots were generated using the R package Gviz[127]. For all manipulations of genomic intervals (resizing, overlaps, distance between regions etc.), the Bioconductor package GenomicRanges was used[128].
