# Supplementary material for: Evidence that direct inhibition of transcription factor binding is the prevailing mode of gene and repeat repression by DNA methylation
Source: Nat Genet. 2022 Dec 5;54(12):1895–906. doi: 10.1038/s41588-022-01241-6 (PMC9729108; doi:10.1038/s41588-022-01241-6)
Supplement: Supplementary file 2 — Reporting Summary [file 41588_2022_1241_MOESM2_ESM.pdf]

nature portfolio

Corresponding author(s): Dirk Schübeler

Last updated by author(s): Sep 26, 2022

Reporting Summary

Nature Portfolio wishes to improve the reproducibility of the work that we publish. This form provides structure for consistency and transparency in reporting. For further information on Nature Portfolio policies, see our Editorial Policies and the Editorial Policy Checklist.

Statistics

For all statistical analyses, confirm that the following items are present in the figure legend, table legend, main text, or Methods section.

n/a	Confirmed	
☐	☒	The exact sample size (*n*) for each experimental group/condition, given as a discrete number and unit of measurement
☐	☒	A statement on whether measurements were taken from distinct samples or whether the same sample was measured repeatedly
☐	☒	The statistical test(s) used AND whether they are one- or two-sided<br>*Only common tests should be described solely by name; describe more complex techniques in the Methods section.*
☐	☒	A description of all covariates tested
☐	☒	A description of any assumptions or corrections, such as tests of normality and adjustment for multiple comparisons
☐	☒	A full description of the statistical parameters including central tendency (e.g. means) or other basic estimates (e.g. regression coefficient) AND variation (e.g. standard deviation) or associated estimates of uncertainty (e.g. confidence intervals)
☐	☒	For null hypothesis testing, the test statistic (e.g. *F*, *t*, *r*) with confidence intervals, effect sizes, degrees of freedom and *P* value noted<br>*Give P values as exact values whenever suitable.*
☒	☐	For Bayesian analysis, information on the choice of priors and Markov chain Monte Carlo settings
☒	☐	For hierarchical and complex designs, identification of the appropriate level for tests and full reporting of outcomes
☐	☒	Estimates of effect sizes (e.g. Cohen's *d*, Pearson's *r*), indicating how they were calculated

Our web collection on statistics for biologists contains articles on many of the points above.

Software and code

Policy information about availability of computer code

Data collection	llumina RTA 1.18.64 (HiSeq 2500) and bcl2fastq2 v2.17, Illumina RTA 2.4.1 (NextSeq 500) and bcl2fastq2 v2.17 or llumina RTA 3.4.5 (NovaSeq 6000) and bcl2fastq2 v2.20 was used for basecalling and demultiplexing
Data analysis	Data analysis was performed using R 3.6.3 and R/Bioconductor packages:, BSgenome.Hsapiens.UCSC.hg19 1.4.0, TxDb.Hsapiens.UCSC.hg19.knownGene v3.2.2, or BSgenome.Mmusculus.UCSC.mm10 1.4.0, TxDb.Mmusculus.UCSC.mm10.knownGene 3.10.0, org.Mm.eg.db 3.13.0, GenomicRanges 1.44.0, QuasR 1.32.0, clusterProfiler 4.0.5, Gviz 1.36.2, limma 3.48.3, samtools 1.9.<br>ATAC-seq reads were trimmed using cutadapt 2.5. Reads were mapped by bowtie (ChIP-seq, ATAC-seq) or hisat2 (RNA-seq) via the R package QuasR using Rbowtie 1.26.0/bowtie 1.2.2 and Rhisat2 1.2.0/hisat2 2.1.0. Macs2 2.1.3.3 was used for peak finding (ChIP-seq), and HOMER 4.11 for motif enrichment analysis (ChIP-seq). Methylation counts were calculated using the qMeth function from QuasR.

For manuscripts utilizing custom algorithms or software that are central to the research but not yet described in published literature, software must be made available to editors and reviewers. We strongly encourage code deposition in a community repository (e.g. GitHub). See the Nature Portfolio guidelines for submitting code & software for further information.

Data

Policy information about availability of data

All manuscripts must include a data availability statement. This statement should provide the following information, where applicable:

- Accession codes, unique identifiers, or web links for publicly available datasets
- A description of any restrictions on data availability
- For clinical datasets or third party data, please ensure that the statement adheres to our policy

Next-generation sequencing data reported in this study have been deposited at the Gene Expression Omnibus with accession number: GSE184470
https://www.ncbi.nlm.nih.gov/geo/query/acc.cgi?acc=GSE184470

In addition, the following public datasets were analyzed that are available in the GEO repository:
RNA-seq:P5 mouse cortex cUhrf1 KO (GSE84550; GSM2241736/ GSM2241739/ GSM2241740) and matching heterozygote (GSE84550; GSM2241735/ GSM2241737). ES cSetdb1 cDnmt1 KO (GSE77781; GSM2059172 and GSM2059173) and matching WT (GSE77781; GSM2059171). E8.5 whole embryos Dnmt1-KO (GSE130735; GSM3752651 to GSM3752653) and matching WT (GSE130735; GSM3752646 to GSM3752648).
WGBS-seq: CA methylation data (GSE47966).

Jaspar2018 motif database used in this study can be accessed online (https://jaspar2018.genereg.net/). The RepeatMasker (http://www.repeatmasker.org) annotation used in this study was downloaded from the UCSC genome annotation database for the Dec. 2011 (GRCm38/mm10) assembly of the mouse genome (ftp://hgdownload.cse.ucsc.edu/goldenPath/mm10/database/rmskOutBaseline.txt.gz).

Human research participants

Policy information about studies involving human research participants and Sex and Gender in Research.

Reporting on sex and gender	*Use the terms sex (biological attribute) and gender (shaped by social and cultural circumstances) carefully in order to avoid confusing both terms. Indicate if findings apply to only one sex or gender; describe whether sex and gender were considered in study design whether sex and/or gender was determined based on self-reporting or assigned and methods used. Provide in the source data disaggregated sex and gender data where this information has been collected, and consent has been obtained for sharing of individual-level data; provide overall numbers in this Reporting Summary. Please state if this information has not been collected. Report sex- and gender-based analyses where performed, justify reasons for lack of sex- and gender-based analysis.*
Population characteristics	*Describe the covariate-relevant population characteristics of the human research participants (e.g. age, genotypic information, past and current diagnosis and treatment categories). If you filled out the behavioural & social sciences study design questions and have nothing to add here, write "See above."*
Recruitment	*Describe how participants were recruited. Outline any potential self-selection bias or other biases that may be present and how these are likely to impact results.*
Ethics oversight	*Identify the organization(s) that approved the study protocol.*

Note that full information on the approval of the study protocol must also be provided in the manuscript.

Field-specific reporting

Please select the one below that is the best fit for your research. If you are not sure, read the appropriate sections before making your selection.

☒ Life sciences ☐ Behavioural & social sciences ☐ Ecological, evolutionary & environmental sciences

For a reference copy of the document with all sections, see nature.com/documents/nr-reporting-summary-flat.pdf

Life sciences study design

All studies must disclose on these points even when the disclosure is negative.

Sample size	For publicly available datasets used in the study, we included all available replicates from the relevant experimental system. For all other samples, at least two biological replicates were performed to assure reproducibility, in line with accepted practice in the genomics field.
Data exclusions	No data was excluded
Replication	All experiments were performed in at least two biological replicates as indicated in the manuscript to assure reproducibility. All replication attempts were successful, all samples are included in the final publication.

Randomization	Randomization was not considered in this cell culture-based study. Comparison were done between treated and/or knockout and control cells.
Blinding	Blinding was not considered for this study. All cells were grown in identical culture conditions (+/- treatment and/or knockout). No subjective measurements were applied.

Reporting for specific materials, systems and methods

We require information from authors about some types of materials, experimental systems and methods used in many studies. Here, indicate whether each material, system or method listed is relevant to your study. If you are not sure if a list item applies to your research, read the appropriate section before selecting a response.

Materials & experimental systems

n/a	Involved in the study
☐	☒ Antibodies
☐	☒ Eukaryotic cell lines
☒	☐ Palaeontology and archaeology
☒	☐ Animals and other organisms
☒	☐ Clinical data
☒	☐ Dual use research of concern

Methods

n/a	Involved in the study
☐	☒ ChIP-seq
☒	☐ Flow cytometry
☒	☐ MRI-based neuroimaging

Antibodies

Antibodies used

Mouse targets; Vendor; catalog number; dilutions for Western blotting (WB) or ChIP indicated
1. MBD1; Santa Cruz, sc25261 (B5); 1/200 for WB
2. MBD2; Abcam, ab188474; 1/1000 for WB
3. MBD3; Abcam, ab157464; 1/2000 for WB
4. MBD4; Santa Cruz, sc365974; 1/250 for WB
5. MeCP2; Sigma, M7443; 1/1000 for WB
6. CREB1; Santa Cruz, sc377154X; 1/2000 for WB and 5 µg for ChIP
7. POL2; Santa Cruz, sc899X and Abcam, ab817; 5 µg for ChIP
8. ONECUT1; R&D systems, AF6277; 5 µg for ChIP
9. LAMIN; Abcam, ab16048; 1/2000 for WB
10. HISTONE 3; Cell signaling 9715; 1/1000 for WB
11. β-ACTIN; Cell signaling 3700S; 1/2000 for WB

Human targets; Vendor; catalog number; dilutions for Western blotting (WB) indicated
12. MBD1 Abcam, ab108510; 1/500 for WB
13. MBD2 Abcam, ab188474; 1/1000 for WB
14. MBD3 Abcam, ab157464; 1/2000 for WB
15. MBD4 Bethyl laboratories, A301-634AM; 1/1000 for WB
16. MeCP2 Abcam, ab253197; 1/1000 for WB
17. β-ACTIN Cell signaling, 3700S; 1/2000 for WB

Validation

All antibodies validated by manufacturers:
1. Antibody detects a band of expected molecular weight in a Western blot lane with whole cell lysate of HEK293T over expressing mouse MBD1 (manufacturer's website; https://www.scbt.com/p/mbd1-antibody-b-5).
2. Knockout validated in HAP1 cells (manufacturer's website; https://www.abcam.com/mbd2-antibody-epr18361-ab188474.html).
3. Knockout validated in HAP1 cells (manufacturer's website; https://www.abcam.com/mbd3-antibody-epr9913-chip-grade-ab157464.html).
4. Detection of a band at the expected molecular weight in a Western blot lane with SK-MEL nuclear extracts (manufacturer's website; https://www.scbt.com/p/mbd4-antibody-a-8) and validated by knockdown experiments in PMID: 32850324.
5. Validated by manufacturer in Jurkat cells (manufacturer's website; https://www.sigmaaldrich.com/CH/de/product/sigma/m7443) and others (PMID: 23770565).
6. Antibody detects a band of expected molecular weight in a Western blot lane with whole cell lysate of HEK293T over expressing mouse CREB1 (manufacturer's website; https://www.scbt.com/p/creb-1-antibody-d-12).
7. sc899X: Western blot analysis of Pol II expression in A-431, AT-3 and A-673 nuclear extracts (manufacturer's website; https://www.scbt.com/). Ab817: Discontinued antibody. Information not available. (manufacturer's website; https://www.abcam.com/rna-polymerase-ii-ctd-repeat-ysptsps-antibody-8wg16-chip-grade-ab817.html)
8. Detects human HNF6/
ONECUT1 in direct ELISAs and Western blots. In direct ELISAs, approximately 5% crossreactivity with recombinant human ONECUT2 is observed (manufacturer's website; https://www.rndsystems.com/products/human-hnf-6-onecut1-antibody_af6277).
9. Knockout validated in HAP1 cells (manufacturer's website; https://www.abcam.com/lamin-b1-antibody-nuclear-envelope-marker-ab16048.html)
10. Histone H3 Antibody detects endogenous levels of total histone H3 protein. This antibody does not cross-react with other histones (manufacturer's website; cellsignal.com/products/primary-antibodies/histone-h3-antibody/9715).
11. Mouse mAb detects endogenous levels of total β-actin protein. Due to the high sequence identity between the cytoplasmic actin isoforms, β-actin and cytoplasmic γ-actin, this antibody may cross-react with cytoplasmic γ-actin. It does not cross-react with α-

skeletal, α-cardiac, α-vascular smooth, or γ-enteric smooth muscle isoforms (manufacturer's website; https://www.cellsignal.com/products/primary-antibodies/b-actin-8h10d10-mouse-mab/3700)
12. Knockout validated in HAP1 cells (manufacturer's website; https://www.abcam.com/mbd1-antibody-epr3564-ab108510.html)
13. Knockout validated in HAP1 cells (manufacturer's website; https://www.abcam.com/mbd2-antibody-epr18361-ab188474.html)
14. Knockout validated in HAP1 cells (manufacturer's website; https://www.abcam.com/mbd3-antibody-epr9913-chip-grade-ab157464.html).
15. Detection of human MBD4 by Western blot and immunoprecipitation using whole cell lysate of HeLa cells (manufacturer's website; https://www.thermofisher.com/antibody/product/MBD4-Antibody-Polyclonal/A301-634A)
16. Knockout validated in HAP1 cells (manufacturer's website; https://www.abcam.com/mecp2-antibody-epr23201-3-ab253197.html)
17. Mouse mAb detects endogenous levels of total β-actin protein. Due to the high sequence identity between the cytoplasmic actin isoforms, β-actin and cytoplasmic γ-actin, this antibody may cross-react with cytoplasmic γ-actin. It does not cross-react with α-skeletal, α-cardiac, α-vascular smooth, or γ-enteric smooth muscle isoforms (manufacturer's website; https://www.cellsignal.com/products/primary-antibodies/b-actin-8h10d10-mouse-mab/3700)

Eukaryotic cell lines

Policy information about cell lines and Sex and Gender in Research

Cell line source(s)

The Ngn2 expression cassette was integrated into HA36 mouse ES cells (mixed 129-C57Bl/6 strain). HA36 mouse ES cells were published previously (PMID: 21964573). No commercial source available. All subsequent genotypes (DNMT-TKO or MBD-QKO cells) were generated using this cell line. Subsequently, the IAPLTR1a reporter cassette was integrated into WT and DNMT-TKO cells. The HEK293 MBD-QKO cell line was generated from HEK293 obtained from ATCC® (CRL-1573™). Cell lines available upon request.

Authentication

Genotype of all cell lines was tested at the level of DNA sequence. Knockout cell lines were additionally validated at the level of protein (Western blotting).

Mycoplasma contamination

Cell lines were tested negative for mycoplasma contamination

Commonly misidentified lines
(See ICLAC register)

No commonly misidentified cell lines were used in the study

ChIP-seq

Data deposition

☒ Confirm that both raw and final processed data have been deposited in a public database such as GEO.

☒ Confirm that you have deposited or provided access to graph files (e.g. BED files) for the called peaks.

Data access links
May remain private before publication.

https://www.ncbi.nlm.nih.gov/geo/query/acc.cgi?acc=GSE184470 (All sequencing data, including ChIP-seq)

Files in database submission

For each ChIP-seq sample, the GEO entry contains the following files: the rawdata (fastq format) and a file with alignment density per 100 bp in the mouse mm10 (wig file):
fastq files:
TKO_creb_input_r2_1.fastq.gz
TKO_creb_input_r2_2.fastq.gz
TKO_creb_input_r3_1.fastq.gz
TKO_creb_input_r3_2.fastq.gz
TKO_CREB_r1_1.fastq.gz
TKO_CREB_r1_2.fastq.gz
TKO_CREB_r2_1.fastq.gz
TKO_CREB_r2_2.fastq.gz
TKO_CREB_r3_1.fastq.gz
TKO_CREB_r3_2.fastq.gz
TKO_hnf6_1_r1_1.fastq.gz
TKO_hnf6_1_r1_2.fastq.gz
TKO_hnf6_2_r2_1.fastq.gz
TKO_hnf6_2_r2_2.fastq.gz
TKO_hnf6_input_1_r1_1.fastq.gz
TKO_hnf6_input_1_r1_2.fastq.gz
TKO_hnf6_input_2_r2_1.fastq.gz
TKO_hnf6_input_2_r2_2.fastq.gz
TKO_Pol2_ab_2_1.fastq.gz
TKO_Pol2_ab_2_2.fastq.gz
TKO_pol2_input_1_1.fastq.gz
TKO_pol2_input_1_2.fastq.gz
TKO_pol2_input_2_1.fastq.gz
TKO_pol2_input_2_2.fastq.gz
TKO_Pol2_sc_1_1.fastq.gz
TKO_Pol2_sc_1_2.fastq.gz
WT_creb_input_r2_1.fastq.gz

WT_creb_input_r2_2.fastq.gz
WT_creb_input_r3_1.fastq.gz
WT_creb_input_r3_2.fastq.gz
WT_CREB_r1_1.fastq.gz
WT_CREB_r1_2.fastq.gz
WT_CREB_r2_1.fastq.gz
WT_CREB_r2_2.fastq.gz
WT_CREB_r3_1.fastq.gz
WT_CREB_r3_2.fastq.gz
WT_hnf6_2_r1_1.fastq.gz
WT_hnf6_2_r1_2.fastq.gz
WT_hnf6_2_r2_1.fastq.gz
WT_hnf6_2_r2_2.fastq.gz
WT_hnf6_input_2_r1_1.fastq.gz
WT_hnf6_input_2_r1_2.fastq.gz
WT_hnf6_input_2_r2_1.fastq.gz
WT_hnf6_input_2_r2_2.fastq.gz
WT_Pol2_ab_2_1.fastq.gz
WT_Pol2_ab_2_2.fastq.gz
WT_pol2_input_1_1.fastq.gz
WT_pol2_input_1_2.fastq.gz
WT_pol2_input_2_1.fastq.gz
WT_pol2_input_2_2.fastq.gz
WT_Pol2_sc_1_1.fastq.gz
WT_Pol2_sc_1_2.fastq.gz

wig files:
TKO_creb_input_r2.wig.gz
TKO_creb_input_r3.wig.gz
TKO_CREB_r1.wig.gz
TKO_CREB_r2.wig.gz
TKO_CREB_r3.wig.gz
TKO_hnf6_1_r1.wig.gz
TKO_hnf6_2_r2.wig.gz
TKO_hnf6_input_1_r1.wig.gz
TKO_hnf6_input_2_r2.wig.gz
TKO_Pol2_ab_2.wig.gz
TKO_pol2_input_1.wig.gz
TKO_pol2_input_2.wig.gz
TKO_Pol2_sc_1.wig.gz
WT_creb_input_r2.wig.gz
WT_creb_input_r3.wig.gz
WT_CREB_r1.wig.gz
WT_CREB_r2.wig.gz
WT_CREB_r3.wig.gz
WT_hnf6_2_r1.wig.gz
WT_hnf6_2_r2.wig.gz
WT_hnf6_input_2_r1.wig.gz
WT_hnf6_input_2_r2.wig.gz
WT_Pol2_ab_2.wig.gz
WT_pol2_input_1.wig.gz
WT_pol2_input_2.wig.gz
WT_Pol2_sc_1.wig.gz

Genome browser session
(e.g. UCSC)

The following files can be uploaded (all at once) to the UCSC genome browser by pasting all (mm10) URLs into "Paste URLs or data" in "add custom tracks".
for mm10:
http://www.fmi.ch/groupdata/gschub/mbd_paper/TKO_creb_input_r2.bw
http://www.fmi.ch/groupdata/gschub/mbd_paper/TKO_creb_input_r3.bw
http://www.fmi.ch/groupdata/gschub/mbd_paper/TKO_CREB_r1.bw
http://www.fmi.ch/groupdata/gschub/mbd_paper/TKO_CREB_r2.bw
http://www.fmi.ch/groupdata/gschub/mbd_paper/TKO_CREB_r3.bw
http://www.fmi.ch/groupdata/gschub/mbd_paper/TKO_hnf6_1_r1.bw
http://www.fmi.ch/groupdata/gschub/mbd_paper/TKO_hnf6_2_r2.bw
http://www.fmi.ch/groupdata/gschub/mbd_paper/TKO_hnf6_input_1_r1.bw
http://www.fmi.ch/groupdata/gschub/mbd_paper/TKO_hnf6_input_2_r2.bw
http://www.fmi.ch/groupdata/gschub/mbd_paper/TKO_Pol2_ab_2.bw
http://www.fmi.ch/groupdata/gschub/mbd_paper/TKO_pol2_input_1.bw
http://www.fmi.ch/groupdata/gschub/mbd_paper/TKO_pol2_input_2.bw
http://www.fmi.ch/groupdata/gschub/mbd_paper/TKO_Pol2_sc_1.bw
http://www.fmi.ch/groupdata/gschub/mbd_paper/WT_creb_input_r2.bw
http://www.fmi.ch/groupdata/gschub/mbd_paper/WT_creb_input_r3.bw
http://www.fmi.ch/groupdata/gschub/mbd_paper/WT_CREB_r1.bw
http://www.fmi.ch/groupdata/gschub/mbd_paper/WT_CREB_r2.bw
http://www.fmi.ch/groupdata/gschub/mbd_paper/WT_CREB_r3.bw
http://www.fmi.ch/groupdata/gschub/mbd_paper/WT_hnf6_2_r1.bw
http://www.fmi.ch/groupdata/gschub/mbd_paper/WT_hnf6_2_r2.bw
http://www.fmi.ch/groupdata/gschub/mbd_paper/WT_hnf6_input_2_r1.bw
http://www.fmi.ch/groupdata/gschub/mbd_paper/WT_hnf6_input_2_r2.bw
http://www.fmi.ch/groupdata/gschub/mbd_paper/WT_Pol2_ab_2.bw
http://www.fmi.ch/groupdata/gschub/mbd_paper/WT_pol2_input_1.bw
http://www.fmi.ch/groupdata/gschub/mbd_paper/WT_pol2_input_2.bw
http://www.fmi.ch/groupdata/gschub/mbd_paper/WT_Pol2_sc_1.bw

Methodology

Replicates

Between 2 and 3 biological replicates were performed per cell type and condition (indicated).

Sequencing depth

All ChIP-seq samples (Hnf6 aka Onecut1) were sequenced as paired-end 2x75mers (total and uniquely mapped reads are given below, fields separated by a whitespace character):
"Sample Name" "Total Reads" "Uniquely Mapped Reads"
WT_CREB_r1 108389624 46085768
WT_CREB_r2 97271850 40676254
WT_CREB_r3 100981376 41220880
TKO_CREB_r1 102427000 42991210
TKO_CREB_r2 105076746 41285646
TKO_CREB_r3 92993568 38183720
WT_creb_input_r2 113777952 41126618
WT_creb_input_r3 116614236 40848102
TKO_creb_input_r2 109547348 36229328
TKO_creb_input_r3 106700818 37638118
WT_pol_input_1 100355844 41250202
WT_pol_input_2 86833066 37029866
TKO_pol_input_1 65684696 30455774
TKO_pol_input_2 104464314 41903034
WT_Pol2_sc_1 123129736 45575514
WT_Pol2_ab_2 122600886 47719724
TKO_Pol2_sc_1 126896270 52138490
TKO_Pol2_ab_2 125915562 52190226
WT__hnf6_input_2_r1 31014784 12557754
TKO__hnf6_input_r1 33990900 13782140
WT__hnf6_r1 28242368 17701666
TKO__hnf6_r1 26775116 14335318
WT__Hnf6_r2 32215082 14203922
TKO__Hnf6_r2 33017608 14533444
WT__hnf6_input_r2 31342120 11699790
TKO__hnf6_input_r2 30001954 12522556

Antibodies

CREB1 (Santa Cruz, sc377154X);
POL2 (Santa Cruz, sc899X and Abcam, ab817);
ONECUT1 (R&D systems AF6277)

Peak calling parameters

For TF ChIP–seq data, peaks were called using macs2 version 2.1.3.3 on the bam files of the IP samples using callpeak with parameters -t IP.bam --f BAMPE -g mm -q 0.05. No control was used. CREB1 IP replicates two and three (both WT and DNMT-TKO) were paired with their matching input samples, whereas the first IP replicates were matched with the second input replicates, as no matching input samples had been generated. Only peaks with an enrichment (IP vs input) of at least two-fold in at least two replicates were retained for further analysis.

Data quality	ChIP-seq sample quality was assessed using the following criteria:<br>- technical quality (sufficient sequencing depth and unique-hit mapping rates)<br>- reproducibility (high Pearsons's correlation coefficient on the level of peaks).
Software	Illumina RTA 2.4.1 (NextSeq 500) and bcl2fastq2 v2.17 was used for basecalling and demultiplexing.<br>Analysis was performed using R version 4.1.1 and R/Bioconductor packages: QuasR 1.32.0, Rbowtie 1.26.0, Rhisat2 1.2.0.<br>Peak calling: macs2 version 2.1.3.3
