# Supplementary material for: Evidence that direct inhibition of transcription factor binding is the prevailing mode of gene and repeat repression by DNA methylation
Source: Nat Genet. 2022 Dec 5;54(12):1895–906. doi: 10.1038/s41588-022-01241-6 (PMC9729108; doi:10.1038/s41588-022-01241-6)

Unprocessed Western blot images from Extended Data Figure 1b can be found in Source Data of Figure 1

Unprocessed Western blot images from Extended Data Figure 1f

MBD1

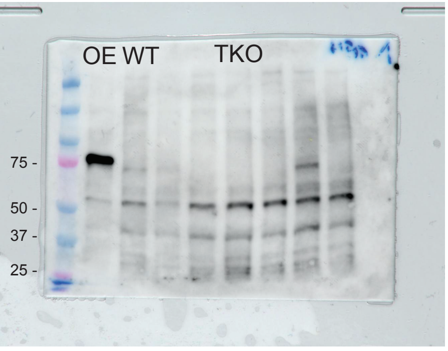

actin

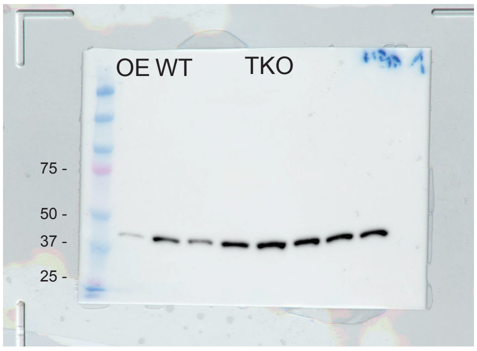

MBD2

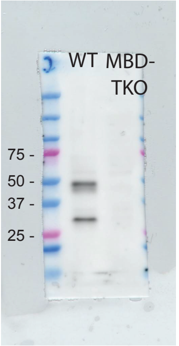

lamin

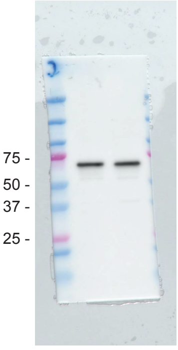

MeCP2

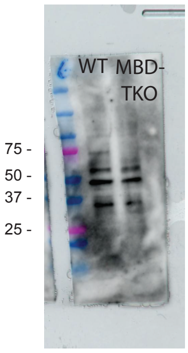

lamin

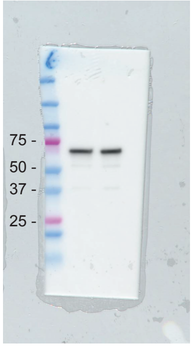

MBD4

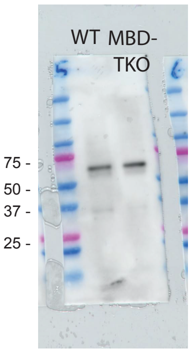

lamin

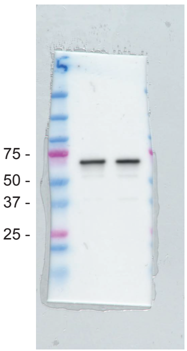

Unprocessed Western blot images from Extended Data Figure 1g

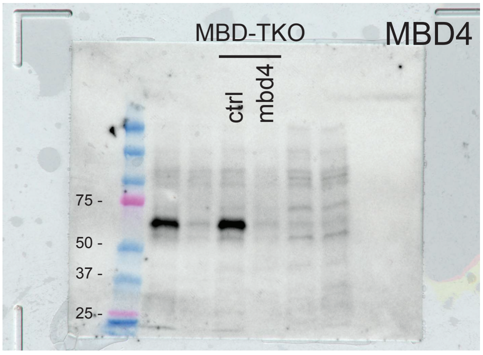

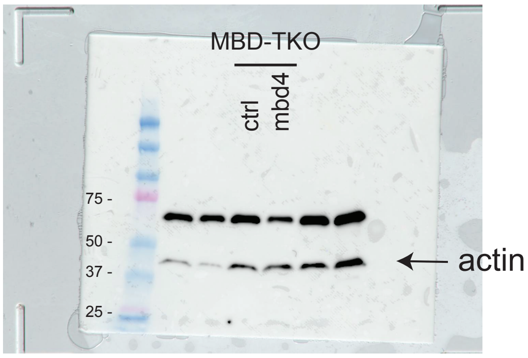

Supplement: Source Data Extended Data Fig. 1 — Unprocessed immunoblots. [file 41588_2022_1241_MOESM5_ESM.pdf]
