# Supplementary material for: Evidence that direct inhibition of transcription factor binding is the prevailing mode of gene and repeat repression by DNA methylation
Source: Nat Genet. 2022 Dec 5;54(12):1895–906. doi: 10.1038/s41588-022-01241-6 (PMC9729108; doi:10.1038/s41588-022-01241-6)

Unprocessed Western blot images from Extended Data Figure 2a

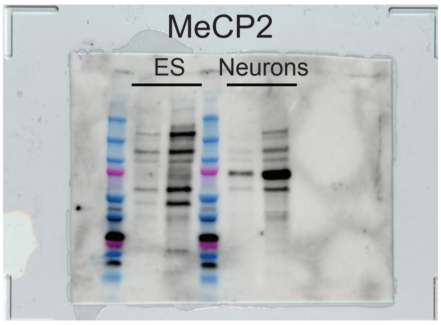

loading control corresponding to MeCP2

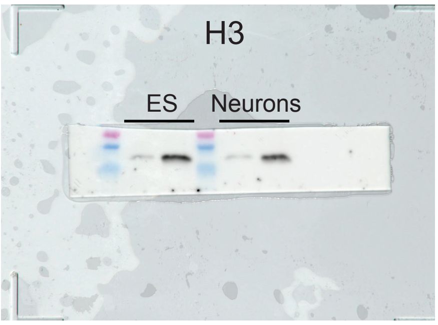

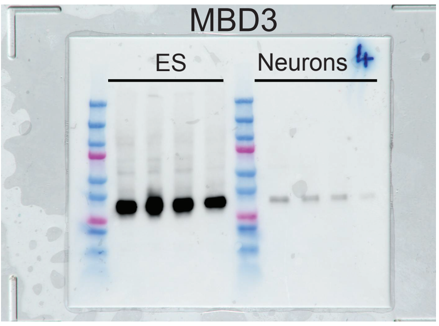

loading control corresponding to MBD3

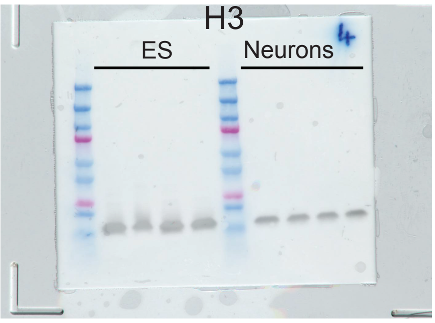

Unprocessed Western blot images from Extended Data Figure 2b

MBD2

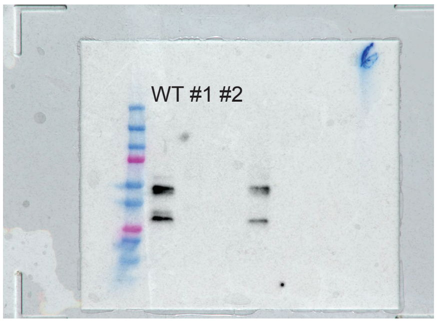

loading control corresponding to MBD2

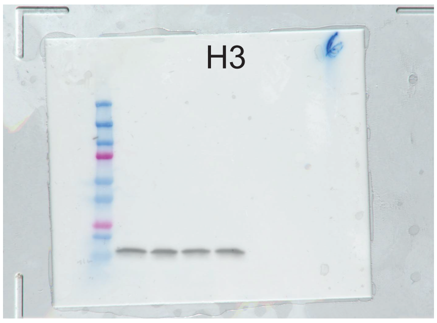

MeCP2

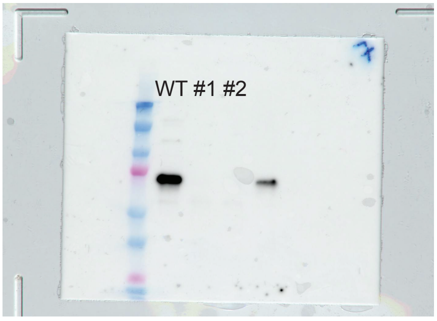

loading control corresponding to MeCP2

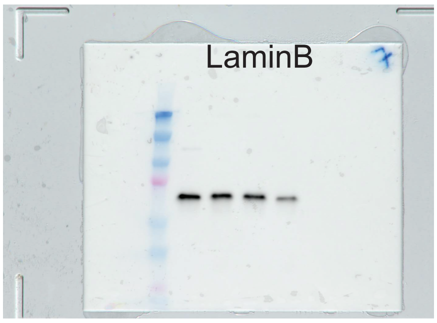

Supplement: Source Data Extended Data Fig. 2 — Unprocessed immunoblots. [file 41588_2022_1241_MOESM6_ESM.pdf]
